# Current Status and Future Prospects of Marine Natural Products (MNPs) as Antimicrobials

**DOI:** 10.3390/md15090272

**Published:** 2017-08-28

**Authors:** Alka Choudhary, Lynn M. Naughton, Itxaso Montánchez, Alan D. W. Dobson, Dilip K. Rai

**Affiliations:** 1 Department of Food Biosciences, Teagasc Food Research Centre Ashtown, Dublin D15 KN3K, Ireland; alka.choudhary@teagasc.ie; 2 School of Microbiology, University College Cork, Western Road, Cork City T12 YN60, Ireland; lynn.naughton@ucc.ie (L.M.N.); a.dobson@ucc.ie (A.D.W.D.); 3 Department of Immunology, Microbiology and Parasitology, Faculty of Science, University of the Basque Country, (UPV/EHU), 48940 Leioa, Spain; itxaso.montanchez@ehu.eus

**Keywords:** antimicrobial, marine natural products (MNPs), secondary metabolites, antibacterial, antifungal, genome mining

## Abstract

The marine environment is a rich source of chemically diverse, biologically active natural products, and serves as an invaluable resource in the ongoing search for novel antimicrobial compounds. Recent advances in extraction and isolation techniques, and in state-of-the-art technologies involved in organic synthesis and chemical structure elucidation, have accelerated the numbers of antimicrobial molecules originating from the ocean moving into clinical trials. The chemical diversity associated with these marine-derived molecules is immense, varying from simple linear peptides and fatty acids to complex alkaloids, terpenes and polyketides, etc. Such an array of structurally distinct molecules performs functionally diverse biological activities against many pathogenic bacteria and fungi, making marine-derived natural products valuable commodities, particularly in the current age of antimicrobial resistance. In this review, we have highlighted several marine-derived natural products (and their synthetic derivatives), which have gained recognition as effective antimicrobial agents over the past five years (2012–2017). These natural products have been categorized based on their chemical structures and the structure-activity mediated relationships of some of these bioactive molecules have been discussed. Finally, we have provided an insight into how genome mining efforts are likely to expedite the discovery of novel antimicrobial compounds.

## 1. Introduction

Infectious diseases caused by bacteria, fungi and viruses pose a major threat to public health despite the tremendous progress in human medicine. A dearth in the availability of effective drugs and the on-going threats posed by antimicrobial resistant organisms further worsen the situation particularly in developing countries. Antimicrobial resistance accounts for at least 50,000 deaths each year in Europe and the United States and it is anticipated that drug resistant infections will be responsible for the deaths of 10 million people worldwide by 2050 [1,2]. Continuously evolving antibiotic-resistance of microbial pathogens has raised demands for the development of new and effective antimicrobial compounds [3]. For generations, humans have turned to nature as a source of invaluable medicinal products, where terrestrial and marine organisms traditionally provide the most effective remedies [4,5]. It was only after the discovery of penicillin in 1928 that microbial sources were explored as sources of new therapeutic molecules. Developments in microbial culture techniques and diving expeditions in the 1970s have largely directed the drug discovery program towards the oceans. Combinatorial chemistry developments in the late 1980s further shifted the emphasis of drug discovery efforts from nature to the laboratory bench [6]. Although the unique structural features associated with natural products cannot be matched by any synthetic library they still continue to inspire researchers in the fields of chemistry, biology, and medicine to develop/synthesize more drug-like molecules [7]. Natural products, as the name suggests, are products of secondary metabolism in nature. Traditionally, many natural products were identified as promising candidates for drug development using bioassay-guided investigations, and chemical structure elucidation techniques [5,8]. However, too often this approach led to the re-isolation of known compounds. Advances in sequencing and ‘-omics’ technologies are expediting the identification and development of novel molecules. Today, over 60% of drugs in the market are derived from natural sources [9,10]. Among the 1562 new chemical entities introduced from the period 1981–2014, 21% are naturally derived, 16% are biological macromolecules, 10% constitute the nature mimic entities, 9% are botanical drugs, 6% constitute vaccines and 4% are unaltered natural products [11]. Several small-molecules from natural sources have been approved as antitumor, antibacterial, and antifungal agents over the period 2011–2014 [11]. In this review, we have discussed the roles played by advances in genomic sequencing and ‘-omics’ technologies in expediting the identification and development of novel, antimicrobial marine natural products (MNPs) from biosynthetically “talented” microorganisms of marine origin.

## 2. Marine Natural Products (MNPs)

The ocean covers over 71% of the earth’s surface and constitutes more than 90% of the inhabitable space on the planet. An estimated 50–80% of all life on earth resides in the ocean and it is home to 32 out of 33 known animal phyla, 15 of which are exclusively marine [12]. More than 20,000 natural products have been discovered in the marine environment over the past 50 years [13]. Interest in marine natural products (MNPs) based drug discovery is evident from the increase in number of isolated MNPs (from an annual number of approximately 20 in 1984 to an annual number of more than 1000 in 2010) [14,15]. From the continuing progress in the area of MNPs seven approved drugs and 12 agents currently in clinical trials have been discovered [16]. These molecules are either natural products, tailored natural products or are molecules inspired from the structure of natural products [17,18]. Marine organisms largely obtained from shallow-water, tropical ecosystems are the major sources of MNPs. Macroorganisms such as algae, sponges, corals and other invertebrates, as well as microorganisms have also contributed significantly towards the discovery of novel MNPs [19]. Marine invertebrates in particular have proven to be major sources of MNPs in clinical trials [20]. Also, mounting evidence suggests that many of the compounds originally associated with the biomass of macroorganisms such as sponges [21], tunicates [22], molluscs [23] amongst others, are not produced by the organism itself but are synthesized by symbiotic or associated microorganisms, or derive from a diet of prokaryotic microorganisms [24]. Unlike the terrestrial environment, where plants are comparatively richer in secondary metabolites, marine invertebrates and bacteria have yielded substantially more bioactive natural products than marine plants [25].

The total number of approved drugs from the marine environment steadily increased from four in 2010 to seven in 2014 [26,27]. The first U.S. Food and Drug Administration (FDA) approved marine-derived drug cytarabine (Cytosar-U^®^), isolated from the Caribbean sponge *Cryptotheca crypta*, reached the market in 1969 for use as an anticancer drug. Since then, six more marine natural products have moved through clinical trials and have been approved as drugs (one of which is only registered in the European Union), including the analgesic cone snail-derived peptide ziconotide (Prialt^®^), and the anticancer sponge-derived macrolide, eribulin mesylate (Halaven^®^), as well as four other products with anticancer, antiviral and antihypertriglyceridemia activities [27]. Of the 23 most recently identified marine-derived compounds, 21 are in several different stages of the clinical pipeline for use as anticancer agents, while two of them are being assessed for treatment of chronic pain and neurological disorders like schizophrenia and Alzheimer’s disease [7]. In addition, a number of other compounds boasting antibacterial, antidiabetic, antifungal, antiinflammatory and antiviral properties, as well as compounds potentially affecting the nervous system, are currently being investigated for use in clinical settings and thus form part of the preclinical pipeline [27,28,29].

## 3. Chemical Entities in the Preclinical Antimicrobial Pipeline

**Figure 1:**
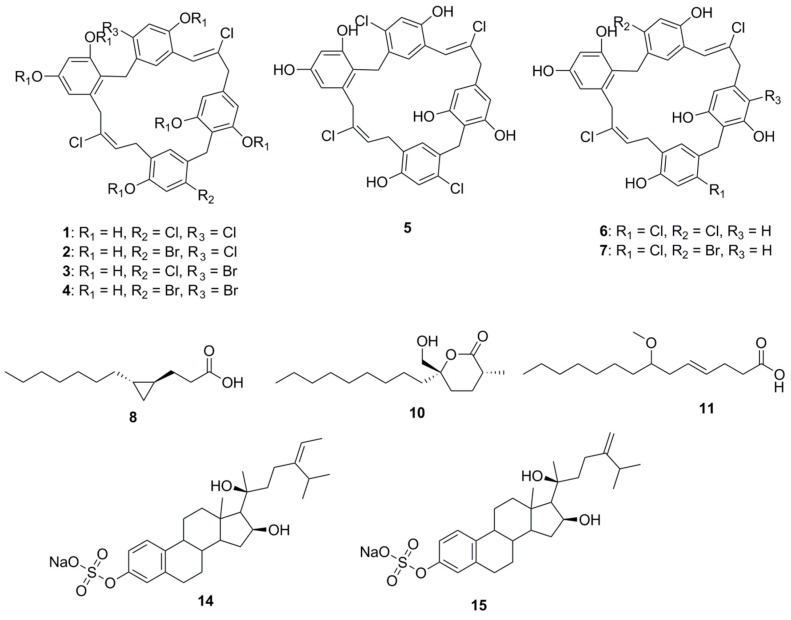
Marine natural products in antimicrobial preclinical studies.

Over the last 5 years, preclinical pharmacology has been undertaken on 262 marine compounds that are presently at various stages of clinical investigations [26]. Herein, we discuss the structural features of some of these molecules (Figure 1) which are currently under investigation for their potential as antimicrobial agents. Where noted in the text, bold numerical values correspond to their associated structures in corresponding figures.

Chrysophaentin A (**1**), a macrocyclic natural product (comprising two polyhalogenated, polyoxygenated ω,ω′-diarylbutene units connected by two ether bonds), was isolated from the chrysophyte alga *Chrysophaeum taylori*. This compound inhibits the growth of clinically relevant Gram-positive bacteria, including methicillin-resistant *Staphylococcus aureus* (MRSA; MIC_50_ 1.5 ± 0.7 μg/mL), multiple drug resistant *S. aureus* (MIC_50_ 1.3 ± 0.4 μg/mL), and Vancomycin-Resistant *Enterococcus faecium* (VREF; MIC_50_ 2.9 ± 0.8 μg/mL). Chrysophaentin A inhibits the GTPase activity of the bacterial cytoskeletal protein FtsZ (IC_50_ value 6.7 μg/mL), and GTP-induced formation of FtsZ protofilaments [30]. Interestingly, this compound was found to be relatively more active among its congeners, Chrysophaentins B–G (**2**–**7**). Analysis of bioactivity of these molecules provided insights into the pharmacophoric features of Chrysophaentins relevant to antimicrobial activity. Phenolic groups in compound **1** were determined to be crucial for activity as a hexaacetate derivative of **1** was found to be inactive at a concentration 25 μg/disk. An approximate 12-fold decrease in the MIC_50_ value of chrysophaentin D (**4**) compared to compound **1** was observed on replacement of chlorine with bromine on rings A and C. The significance of the macrocyclic structure was established following higher MIC_50_ values of Chrysophaentin E (**5**) being observed towards *S. aureus* and MRSA compared to MIC_50_ values observed for the chlorinated cyclic bisdiarylbutene ethers **1** and **6**. However, compound **5** was also found to be inactive toward *E. faecium* and VREF at concentrations as high as 25 μg/mL. In the case of the symmetrically linked dimers **6** and **7**, replacing a chlorine atom on ring C with bromine confers compound **6** with at least three times better activity than compound **7**. Among the tetrachlorinated macrocyles **1** and **6**, compound **1** was found to be 3–5 times more potent than compound **6**, indicating that the position of the ether linkage relative to the 2-butene unit affects activity. In fact, *ortho*-linked chrysophaentin A has been found to be more potent than the *para*-linked chrysophaentin F [31].

A small cyclopropane-containing fatty acid, lyngbyoic acid (**8**), was found to be a major metabolite of the marine cyanobacterium, *Lyngbya* cf. *majuscula*, isolated in Florida [32]. This molecule exerts antimicrobial action by disrupting quorum sensing in *Pseudomonas aeruginosa.* At a concentration of 100 µM, it inhibits the *N*-acyl homoserine lactone (HSL) receptor proteins in the organism (LasR in particular), reducing the expression of important virulence factors in the wild-type strain. The molecules inhibit the response of LasR-based QS reporter plasmids to 3-oxo-C 12-HSL. The AHL-binding site of LasR was not essential to this effect, but competition experiments indicated that compound **8** is likely to have a dual mechanism of action acting both through the AHL-binding site and independently of it. Comparison of compound **8** with related compounds (dodecanoic acid, **9**; malyngolide, **10**; and lyngbic acid, **11**; methyl ester of dodecanoic acid, **12** and butyric acid, **13**) revealed a structure-activity relationship. While compounds **9**, **10** and **11** had a similar potency in pSB1075 compared to **8**, either esterification (**12**) or shortening of the alkyl chain (**13**) reduced activity [32].

Two sulfated sterols, geodisterol-3-*O*-sulfite (**14**) and 29-demethylgeodisterol-3-*O*-sulfite (**15**), were isolated from the marine sponge *Topsentia* sp. These sulfated sterols demonstrated reverse efflux pump-mediated fluconazole resistance. They enhanced the activity of fluconazole in a *Saccharomyces cerevisiae* strain overexpressing the *Candida albicans* efflux pump MDR1, and in a fluconazole-resistant *C. albicans* clinical isolate known to overexpress MDR1. No activity for non-sulfated sterol in fluconazole-resistance reversal assay had been observed highlighting the relevance of sulfate group for MDR1 inhibition and synergy with fluconazole. Investigation of the geodisterols had provided insight into the clinical utility of combining efflux pump inhibitors with current antifungals to combat the resistance associated with opportunistic fungal infections caused by *C. albicans* [33].

In the following sections, we present a systematic overview of the marine natural products which have gained the attention of chemists and biologists over the last five years (2012–2017) as potential antimicrobial agents. The molecules are categorized according to their chemical class based on their associated structural units. Pharmacophoric features responsible for antimicrobial activity are also discussed. Table 1 lists MNPs and describes their antimicrobial potential in terms of MICs and zone of inhibition.

## 4. Alkaloids

**Figure 2:**
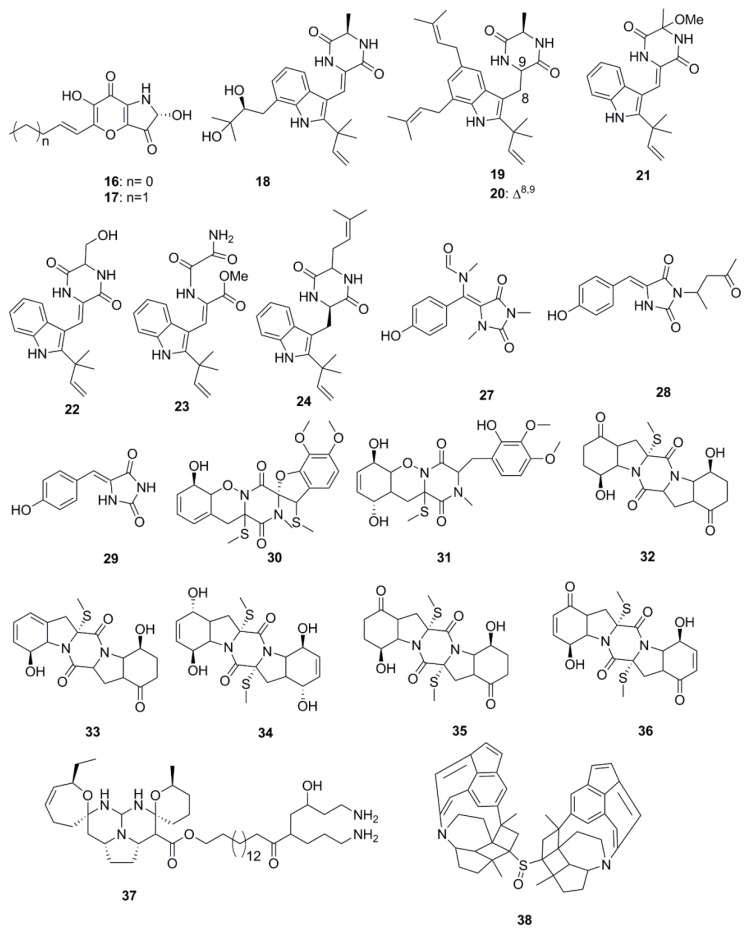

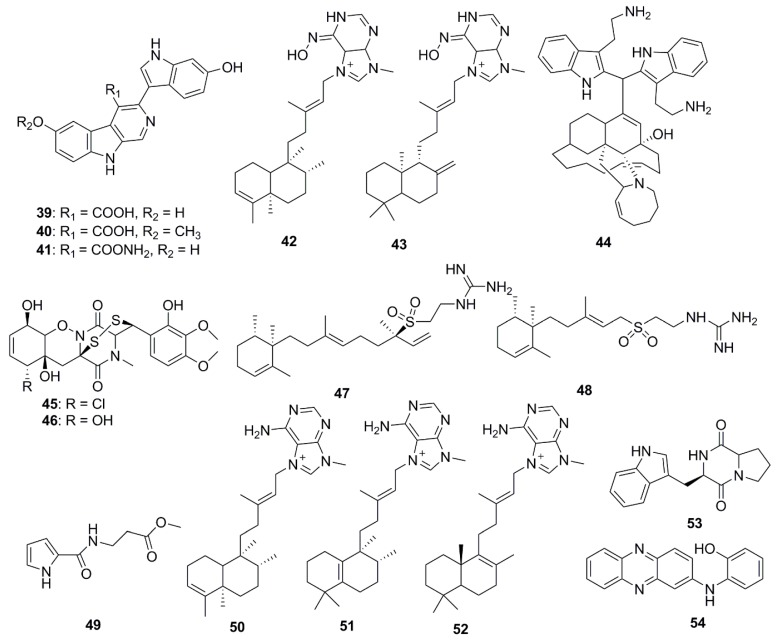
Antimicrobial alkaloids.

Alkaloids constitute the largest number of antimicrobial compounds reported from marine species (Figure 2). Polyoxygenated dihydropyrano[2,3-*c*]pyrrole-4,5-dione derivatives, pyranonigrin A (**16**) and F (**17**) were isolated and identified from *Penicillium brocae* MA-231, which is an endophytic fungus obtained from the fresh tissue of the marine mangrove plant *Avicennia marina*. These compounds possess a wide array of antimicrobial activities against a human-, aqua-, and plant-pathogens [34]. Indole diketopiperazine compounds (**18**–**21**) identified from the culture extract of *Eurotium cristatum* EN-220 (endophytic fungus species obtained from the marine alga *Sargassum thunbergii*) possess antimicrobial activities. While rubrumazine B (**18**) exhibited moderate activity (MIC 64 µg/mL) against the plant-pathogenic fungus *Magnaporthe grisea*, echinulin (**19**), dehydroechinulin (**20**) and variecolorin H (**21**) showed mild activity (MIC 256 µg/mL) against the human pathogen *S. aureus*. This particular trend in antimicrobial activity led to an assumption that indole diketopiperazine alkaloids formed by condensation of a tryptophan residue with a second amino acid such as l-alanine might have low relevance to the observed antimicrobial activity [35]. In a separate study by Du et al., a set of indole alkaloids including echinulin (**19**), cristatumin A (**22**), cristatumin D (**23**) and tardioxopiperazine A (**24**) were found to be active against *Escherichia coli* and *S. aureus* bacteria but were unable to inhibit the growth of the plant-pathogenic fungi, *Alternaria brassicae*, *Valsa mali*, *Physalospora obtuse*, *Alternaria solania*, and *Sclerotinia miyabeana*. The antibacterial activity of cristatumin A (**22**) appeared to be related to the serine residue in the 2,5-diketopiperazine moiety compared to that of neoechinulin A (**25**), which contained an alanine residue. The structural difference between isoechinulin A (**26**) and tardioxopiperazine A (**24**) was found at the C-8/C-9 position, where the single bond between C-8/C-9 in compound **24** was essential for its antibacterial activity [36].

*N*-alkylated hydantoin alkaloids, such as hemimycalins A (**27**) and B (**28**) and (*Z*)-5-(4-hydroxybenzylidene)imidazolidine-2,4-dione (**29**), isolated from the Red Sea sponge, *Hemimycale arabica* have previously demonstrated antimicrobial activity against *E. coli* and *C albicans* but were found to be inactive against *S. aureus* [37]. Peniciadametizine A (**30**), a dithiodiketopiperazine derivative possessing a unique spiro [furan-2,7′-pyrazino[1,2-b][1,2]oxazine] skeleton, and its highly oxygenated analogue, peniciadametizine B (**31**) were isolated from *Penicillium adametzioides* AS-53, a fungus obtained from an unidentified marine sponge collected in Wenchang, Hainan, China. These compounds exhibited selective activities against *A. brassicae* but were inactive against bacteria (*Aeromonas hydrophilia*, *Edwardsiella tarda*, *E. coli*, *S. aureus*, *Vibrio alginolyticus*, *V. anguillarum*, *V. parahaemolyticus*, and *V. harveyi*), and plant-pathogenic fungi (*Colletotrichum gloeosporioides*, *Fusarium graminearum*, and *Gaeumannomyces graminis*) [38]. The antimicrobial sulfide diketopiperazine derivatives, penicibrocazines A–E (**32**–**36**), were isolated from a culture extract of *Penicillium brocae* MA-231, an endophytic fungus obtained from the fresh tissue of the marine mangrove plant *Avicennia marina*. Penicibrocazines B–E (compounds **33**–**36** shown in Figure 2) have demonstrated a wide spectrum of activity against several human, aquatic and plant-pathogenic microbes including *S. aureus*, *Micrococcus luteus* and *Gaeumannomyces graminis* but were inactive against *Eromonas hydrophilia*, *E. coli*, *V. harveyi*, *V. parahaemolyticus* and the plant-pathogenic fungi, *A. brassicae*, *Colletotrichum gloeosporioides* and *Fusarium graminearum*. Penicibrocazine A (**32**) was inactive against this entire array of microorganisms. The double bonds at C-6 and C-6′ increase the activity against *S. aureus* (**34** vs. **32**). In addition, a higher number of S-methyl groups is likely to strengthen their activity against *G. graminis* (**35** vs. **32**), while keto groups at C-5/5′ (**36** vs. **34**) are responsible for the enhanced antimicrobial activity [39]. Crambescidin 800 (**37**), a pentacyclic guanidine alkaloid isolated from the sponge *Clathria cervicornis*, has expressed specific inhibitory activity against *Acinetobacter baumannii*, *Klebsiella pneumonia* and *P. aeruginosa* [40].

Xinghaiamine A (**38**), an alkaloid isolated from the marine-derived actinomycete *Streptomyces xinghaiensis* NRRL B24674, has exhibited a broad-spectrum of antibacterial activities against both Gram-negative (*Acinetobacter baumannii*, *P. aeruginosa* and *E. coli*) and Gram-positive pathogens (*S. aureus* and *B. subtilis*). The inhibition of these pathogenic bacteria and clinical MRSA isolates demonstrates the potential of compound **38** to be an effective antibiotic against multi-drug resistant pathogens, particularly *S. aureus* and *A. baumanii*. However, the molecule displayed no obvious antifungal activity against *C. albicans* when tested at concentrations up to 176.64 µM. The sulfoxide moiety present in Xinghaiamine A (**38**) is unusual among the metabolites produced by marine actinomycetes and confers compounds with a broad spectrum of biological activities, including potent antimicrobial activities [41]. Bioassay-directed fractionation performed on the ethyl acetate fraction of an organic extract from the Red Sea sponge *Hyrtios* species yielded the alkaloid compounds, hyrtioerectines D–F (**39**–**41**), which possessed antimicrobial activities against *C. albicans*, *S. aureus* and *P. aeruginosa* but not against *E. coli*. The relatively higher antimicrobial activity exerted by compounds **39** and **41** with respect to compound **40** could be attributed to the presence of diphenolic moieties in their structure. Amidation of the carboxylic moiety in compound **41** exerted a slight effect on activity when compared to compound **39** [42]. The diterpene alkaloids, ageloxime B (**42**) and (−)-ageloxime D (**43**), were isolated from the marine sponge *Agelas mauritiana*, isolated from Yongxing island in the South China Sea. Both compounds (**42** and **43**) were able to inhibit the growth of *C. neoformans*. Compound **42** also exhibited antibacterial activity against *S. aureus* and MRSA [43]. A manzamine alkaloid, i.e., zamamidine D (**44**), was isolated from an Okinawan *Amphimedon* sp. marine sponge. Compound **44** is the first manzamine alkaloid possessing a 2,2′-methylenebistryptamine unit as its aromatic moiety instead of a *β*-carboline unit, which affected growth of both bacteria and fungi [44]. Chemical investigation of the marine-sponge derived fungus *Penicillium adametzioides* AS-53 yielded the bisthiodiketopiperazine derivatives, adametizines A (**45**) and B (**46**), differing in the presence of a chlorine group at C-7 in compound **45** and hydroxyl in compound **46**. The presence of a chlorine atom at C-7 conferred compound **45** with better antibacterial activity than compound **46** [45]. 

Diterpene alkaloids from the sponge *Agelas nakamurai* collected from the Xisha Islands in the South China Sea have demonstrated significant antimicrobial potential in vitro against *S. aureus*, *E. coli*, *Proteusbacillus vulgaris* and *C. albicans*. Iso-agelasidine B (**47**) and (−)-agelasidine C (**48**) exhibited pronounced antifungal activities (MIC 2.34 µg/mL) against *C. albicans* and weakly inhibited bacterial growth. In addition, diterpene alkaloids containing a 9-*N*-methyladeninium unit: iso-agelasine C (**49**), agelasine B (**50**), agelasine J (**51**) and nemoechine G (**52**) also possessed strong antifungal activities (MIC 0.6 µg/mL) against *C. albicans* and weak to moderate antibacterial activities [46]. Brevianamide F (**53**) was isolated from the marine-derived fungus, *Penicillium vinaceum*. This compound demonstrated antimicrobial activity against *S. aureus* and antifungal activity towards *C. albicans* [47]. A new secondary metabolite *N*-(2-hydroxyphenyl)-2-phenazinamine (**54**) was isolated from the saline culture broth of *Nocardia dassonvillei*, a marine actinomycete recovered from a sediment in the Arctic Ocean. This new compound has shown significant antifungal activity against *C. albicans*, with a MIC 64 µg/mL [48].

## 5. Terpenoids

**Figure 3:**
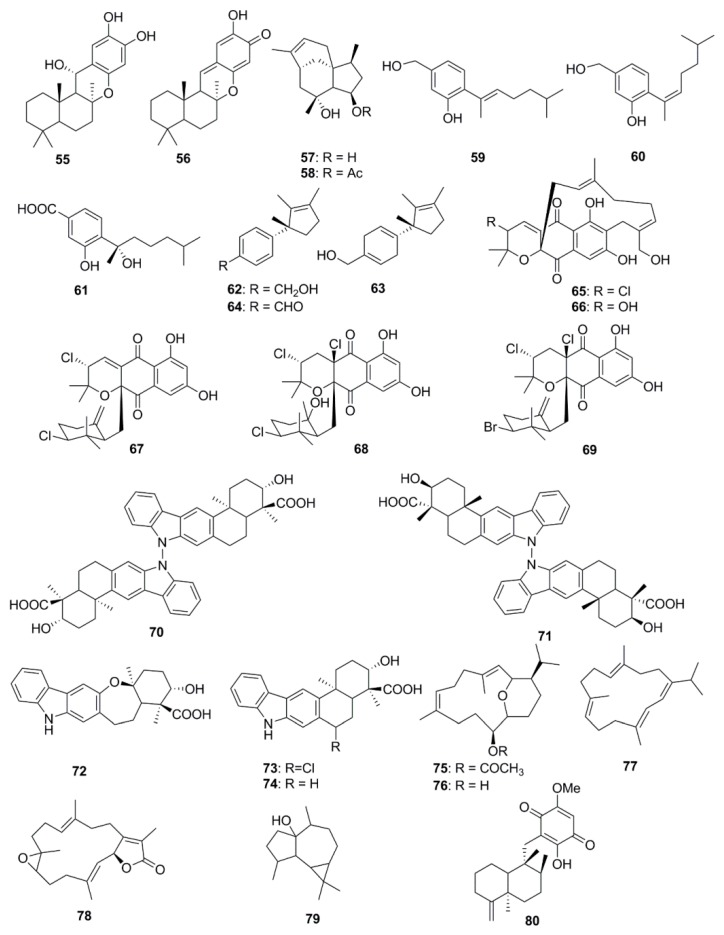
Antimicrobial terpenoids.

As many as 26 different types of terpenoids have been found in marine species (Figure 3). A potent antimicrobial meroterpenoid, puupehenol (**55**) was isolated from the organic extract of a deep-water Hawaiian sponge *Dactylospongia* sp., along with puupehenone (**56**) which has been suggested to be a derivative of puupehenol. These compounds exhibited antibacterial activity towards the Gram-positive bacteria, *S. aureus* and *B. cereus*, but no inhibition was observed against *E. coli* and *P. aeruginosa* [49]. The sesquiterpenes, Penicibilaenes A (**57**) and B (**58**) isolated from *Penicillium bilaiae* MA-267, a fungus obtained from the rhizospheric soil of the mangrove plant *Lumnitzera racemose* possess a tricyclo[6.3.1.0^1,5^]dodecane skeleton, which confers antimicrobial activities to these compounds. Both of these molecules have demonstrated selective activity against *C. gloeosporioides*. Compound **57** proved more active than **58** suggesting that the acetylation of 4-OH enhances the bioactivity of the compound [50]. Sesquiterpenoids including aspergillusene A (**59**), (*Z*)-5-(Hydroxymenthyl)-2-(6′)-methylhept-2′-en-2′-yl)-phenol (**60**) and sydonic acid (**61**) isolated from the sponge-associated fungus, *Aspergillus sydowii* ZSDS1-F6 displayed antimicrobial activities against *Klebsiella pneumonia* and *Aeromonas hydrophila* [51]. Laurene-type sesquiterpenes, 12-hydroxy isolaurene (**62**), 8,11-dihydro-12-hydroxy isolaurene (**63**) and isolauraldehyde (**64**) were isolated from the organic extract of the red alga *Laurencia obtuse* collected off the coast of Jeddah in Saudia Arabia. These compounds exhibited potent activity against the Gram-positive bacteria, *B. subtilis* and *S. aureus*, with compound **64** proving to be the most active (MIC 35 and 27 µg/mL, respectively). Compound **64** also significantly inhibited *C. albicans* (MIC of 70 µg/mL) while, no significant activity against the Gram-negative bacterium *P. aeruginosa* was observed [52]. Napyradiomycins including Napyradiomycin 1 (**65**) and 2 (**66**), Napyradiomycin B2–B4 (**67**–**69**) isolated from marine-derived, *Streptomyces s*trains were found to be active against MRSA [53]. Antibacterial *N*-*N*-coupled indolo-sesquiterpene atropo-diastereomers, dixiamycin A (**70**), dixiamycin B (**71**), oxiamycin (**72**), chloroxiamycin (**73**) and xiamycin A (**74**) were isolated from a marine-derived actinomycete, *Streptomyces* sp. SCSIO 02999. Compounds **70** and **71** were identified as the first examples of naturally occurring atropo-diastereomers containing an unusual *N*-*N*-coupled dimeric indolo-sesquiterpene skeleton and a stereogenic *N*-*N* axis, whilst compound **72** was characterized to contain an unusual seven-membered oxa-ring. Interestingly, the two dimeric compounds **70** and **71** displayed better antibacterial activities than the monomers (compounds **72**–**74**) against four tested strains [54]. Antimicrobial compounds with rare pyrane-based cembranoid structure, sarcotrocheliol acetate (**75**) and sarcotrocheliol (**76**), along with cembranoid, cembrene-C (**77**), sarcophine (**78**), and the aromadendrene sesquiterpenoid, palustrol (**79**) were isolated from the soft coral *Sarcophyton trocheliophorum*. Compounds **75** and **76** displayed significant antibacterial activity, especially against *S. aureus*, *Acinetobacter* sp., and MRSA with MICs ranging from 1.53 to 4.34 µM, while compound **77** demonstrated antifungal activity against *Aspergillus flavus* and *C. albicans* with an MIC of 0.68 µM [55]. A sesquiterpenoid quinone, epi-ilimaquinone (**80**), isolated from the Fijian marine sponge *Hippospongia* sp. was found to possess antibacterial activity against MRSA, wild-type *S. aureus* and VREF and displayed antifungal activity against amphotericin-resistant *C. albicans* [56].

## 6. Lipids

**Figure 4:**
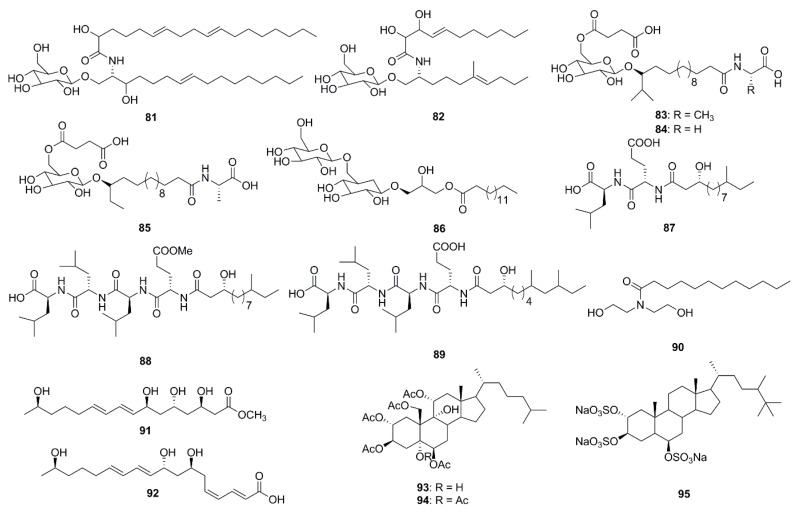
Antimicrobial lipids.

In the past 5 years alone, 15 lipids possessing antibacterial activities were isolated from marine species (Figure 4). Two cerebrosides, penicillosides A (**81**) and B (**82**), were isolated from Red Sea marine-derived fungi and the fungus *Penicillium* species isolated from the tunicate *Didemnum* species. Penicilloside A (compound **81**) displayed antifungal activity towards *C. albicans* displaying an inhibition zone of 23 mm, while Penicilloside A (**82**) possessed antibacterial activities against *S. aureus* and *E. coli* displaying inhibition zones of 19 mm and 20 mm respectively, at 100 µg/disk concentration [57]. Glycolipopeptides-ieodoglucomides A–C (**83**–**85**), and a monoacyldiglycosyl glycerolipid-iedoglycolipid (**86**) isolated from the fermentation broth of the marine sediment-derived bacterium *Bacillus licheniformis* displayed promising antimicrobial activities against Gram-positive and Gram-negative bacteria. Moreover these two pencillosides were also active against the plant pathogenic fungi *Aspergillus niger*, *Rhizoctonia solani*, *Colletotrichum acutatum*, *B. cenerea* and *C. albicans* [58,59]. Gageotetrins A–C (**87**–**89**), fall under a unique class of linear lipopeptides (di- and tetrapeptides) isolated from a marine-derived *Bacillus* were found to be comparatively more active against fungi than to bacteria with MIC values of 0.02–0.04 μM. Moreover, compounds **88** and **89** at a concentration of 0.02 µM displayed potent motility inhibition and lytic activity against the late blight pathogen *Phytophthora capsici*, which causes enormous economic damage in cucumber, pepper, tomato and beans [60]. Lauramide diethanolamine (**90**) was isolated from the marine bacterial strain *Streptomyces* sp. (strain 06CH80). Although this compound (**90**) showed moderate antimicrobial activities against clinical pathogens, its chemical structure is particularly unusual containing a unique carbon skeleton which is different from any other existing antimicrobial agents. This unusual structure provides researchers with the exciting opportunity of exploring various chemical modifications of the compound with the aim of developing of potentially more efficient antimicrobial agents [61]. The unsaturated fatty acids, linieodolides A (**91**) and B (**92**), were isolated from the culture broth of a marine *Bacillus* and the mechanism of their antimicrobial activity was proposed through the inhibition of bacterial fatty acid synthesis [62]. Two highly acetylated steroids, dysiroid A (**93**) and dysiroid B (**94**), were isolated from the marine sponge, *Dysidea* sp. Compounds **93** and **94** showed potent activity against bacterial strains with MICs ranging from 4 to 8 μg/mL [63]. Halistanol sulfate A (**95**) was obtained from the sponge *Petromica ciocalyptoides* and determined to be an effective antibacterial compound against *Streptococcus mutans*. The compound inhibited *S. mutans* biofilm formation by down regulating the expression of the *gtfB*, *gtfC* and *gbpB* virulence genes. Compound **95** inhibited biofilm formation in two *S. mutans* strains at low MIC, but did not inhibit initial colonization by *S. sanguinis*. Such activity is highly desirable in preventative treatments that inhibits pathogenic bacteria via the disruption of biofilm formation without affecting the healthy normal microflora [64].

## 7. Peptides

**Figure 5:**
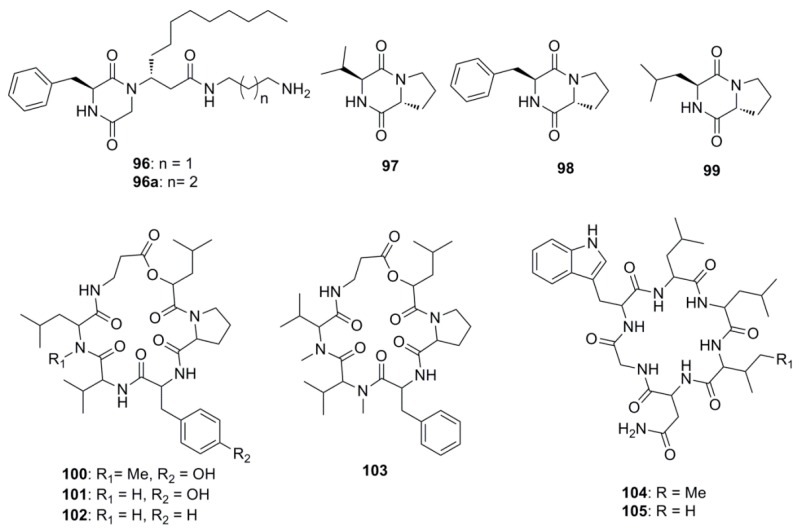
Antimicrobial peptides.

Marine derived antimicrobial compounds of peptide origin are shown in Figure 5. Modified diketopiperazines, rodriguesines A and B (**96** and **96a**) isolated as an inseparable mixture from the ascidian *Didemnum* sp. were found to possess broad antimicrobial activities inhibiting both oral streptococci and pathogenic bacteria. These diketopiperazines are reported to modulate the LuxR-mediated quorum-sensing systems of Gram-negative and Gram-positive bacteria and are considered to influence cell-cell bacterial signaling, offering alternative ways to control biofilms by interfering with microbial communication [64]. Diketopiperazines, cyclo-(l-valyl-d-proline) (**97**) cyclo-(l-phenylalanyl-d-proline (**98**), were isolated from a *Rheinheimera japonica* strain KMM 9513 collected from shores off of the Sea of Japan. These diketopiperazines inhibited the growth of *B. subtilis*, *Enterococcus faecium* and *S. aureus* but were inactive against *E. coli*, *S. epidermidis*, *Xanthomonas* sp. *pv. badrii* and *C. albicans* [65]. Compounds cyclo-(*S*-Proline-*R*-Leucine) (**99**) and (**97**) were isolated from *Bacillus megaterium* LC derived from the marine sponge *Haliclona oculata*. These compounds showed antimicrobial activity at MIC values ranging from 0.005 to 5 µg/mL against Gram-negative bacteria *V. vulnificus* and *V. parahaemolyticus*, together with the Gram-positive bacteria *B. cereus* and *M. luteus* [66]. Cyclohexadepsipeptides of the isaridin class including isaridin G (**100**), desmethylisaridin G (**101**), desmethylisaridin C1 (**102**) and isaridin E (**103**), were identified in the marine bryozoan-derived fungus *Beauveria felina* EN-135. These compounds possessed inhibitory activities against *E. coli* with MICs in the range of 8–64 µg/mL [67]. Desotamide (**104**) and desotamide B (**105**) are cyclic hexapeptides isolated from the marine microbe *Streptomyces scopuliridis* SCSIO ZJ46. When these compounds were explored for their antibacterial potential, notable antibacterial activities against strains of *S. pnuemoniae*, *S. aureus*, and methicillin-resistant *Staphylococcus epidermidis* (MRSE) were observed [68].

## 8. Halogenated Compounds

**Figure 6:**
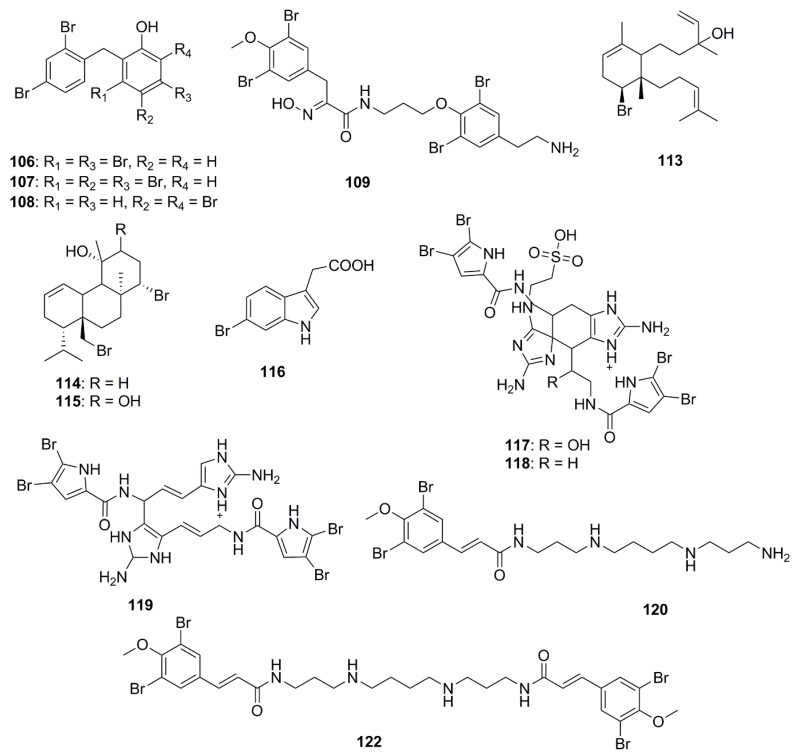
Antimicrobial halogenated compounds.

The chemical structures of halogenated compounds with antimicrobial properties isolated between 2012 and 2017 are presented in Figure 6. Compounds demonstrating a broad spectrum of antibacterial activities were polybrominated diphenyl ethers, 2-(2′,4′-dibromophenoxy)-3,5-dibromophenol (**106**) and 2-(2′,4′-dibromophenoxy)-3,4,5-tribromophenol (**107**) isolated from the marine sponge *Dysidea granulosa*; and 2-(2′,4′-dibromophenoxy)-4,6-dibromophenol (**108**) from *Dysidea* spp. These brominated ethers exhibited in vitro antibacterial activity against MRSA, methicillin-sensitive *Staphylococcus aureus* (MSSA), *E. coli* O157:H7, and *Salmonella.* Structurally compound **106** differed from compound **108** at a bromo-substituted position, and from compound **107** by containing an additional bromo group at the C-4 position. From the structure-activity relationships it was suggested that bromination and para-substitution decreases the antimicrobial activities of bromophenols, except against the human pathogen, *Listeria monocytogenes* [69]. Bromotyrosine-derived alkaloids were isolated by bioassay-guided fractionation of extracts from the Australian marine sponge *Pseudoceratina purpurea*. Aplysamine 8 (**109**) was not found to have any notable activity against *E. coli* or *S. aureus* while hexadellin (**110**), aplysamine 2 (**111**) and 16-debromoaplysamine 4 (**112**) displayed activity against *S. aureus* at concentrations ranging from 125–250 µg/mL [70]. The brominated diterpene, sphaerodactylomelol (**113**) which belongs to the rare dactylomelane family, bromosphaerol (**114**) and 12*R*-hydroxybromosphaerol (**115**) were isolated from cosmopolitan red algae, *Sphaerococcus coronopifolius*, collected in the Atlantic. These compounds inhibited the growth of *S. aureus* but were inactive against *E. coli* and *P. aeruginosa* [71]. A brominated compound, 6-bromoindolyl-3-acetic acid (**116**) displayed varied activities against both Gram-positive and Gram-negative bacteria*: V. harveyi*, *Photobacterium damselae subsp. damselae*. *A. hydrophila*, *S. aureus* and *B. subtilis* [72]. The bromopyrrole alkaloids, nagelamides X–Z (**117**–**119**), isolated from a marine sponge *Agelas* sp. exhibited antimicrobial activity against a range of bacteria and fungi. Compounds **117** and **118** are dimeric bromopyrrole alkaloids with a novel tricyclic skeleton, which consists of spiro-bonded tetrahydrobenzaminoimidazole and aminoimidazolidine moieties, while compound **118** is the first dimeric bromopyrrole alkaloid involving the C-8 position in dimerization [73]. The antibacterial bromotyrosine-derived metabolites, ianthelliformisamines A–C (**120**–**122**), were isolated from the marine sponge *Suberea ianthelliformis*. Compound **120** displayed selective inhibitory activity against *P. aeruginosa* while compound **121** showed little inhibition. The spermine moiety associated with compounds **120** and **122** appeared to be important for activity against *P. aeruginosa*, since replacement of spermine by spermidine in compound **121** reduced the activity significantly. Furthermore, the addition of an extra cinnamyl derivative in compound **122** to the terminal amine of the spermine chain decreased the antibacterial selectivity between *P. aeruginosa* and *S. aureus*; however the observed selectivity may be due to differential cell permeability between the Gram-negative and the Gram-positive bacteria [74]. 

## 9. Polyketides

**Figure 7:**
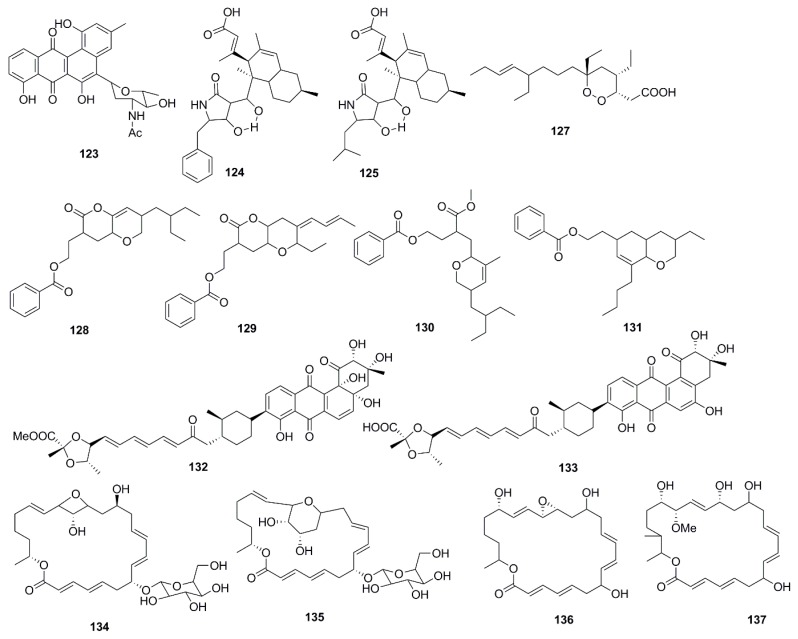

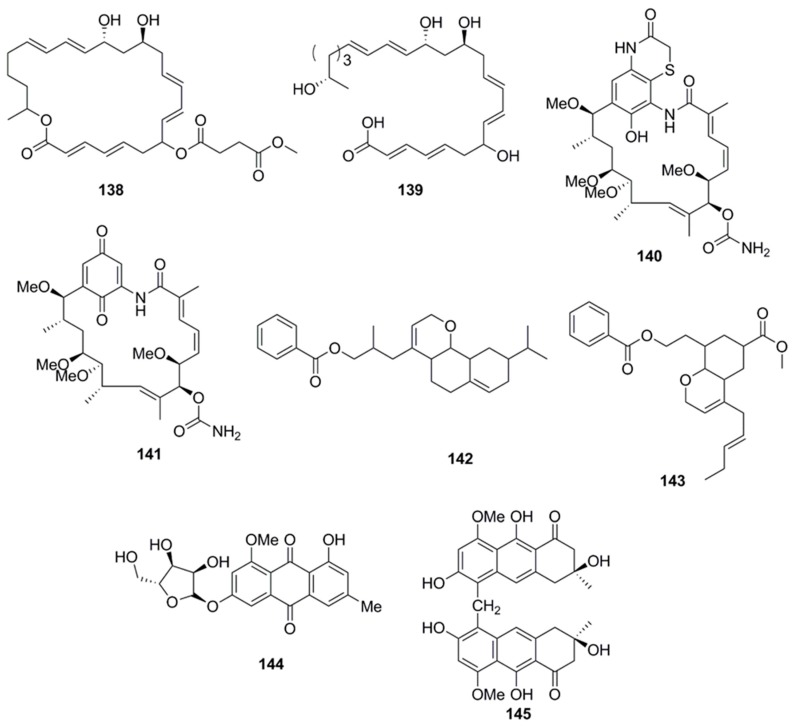
Antimicrobial polyketides.

Antimicrobial marine natural products of polyketide origin are presented in Figure 7. Chemical investigations of cultures of the marine *Streptomyces* sp. 182SMLY led to the discovery of the antimicrobial polycyclic anthraquinone, *N*-acetyl-*N*-demethylmayamycin (**123**), which inhibited the growth of MRSA with a MIC 20 µM but displayed no inhibition against *E. coli* [75]. An unusual antibiotic polyketide with a new carbon skeleton, lindgomycin (**124**), and ascosetin (**125**) extracted from mycelia and culture broth of different *Lindgomycetaceae* strainswere two folds less active against the clinically relevant bacteria *Staphylococcus epidermidis*, *S. aureus*, MRSA, and *Propionibacterium acnes* than standard drug chloramphenicol*.* The antifungal activity of compound **124** and **125** was four times lower than nystatin against the human pathogenic yeast *C. albicans*. Moreover *Xanthomonas campestris* and *Septoria tritici*, were also inhibited by these metabolites but no inhibitory effects against Gram-negative bacteria was observed [76]. Among the polyketide endoperoxides, manadodioxans D (**126**) and E (**127**) isolated from the marine sponge *Plakortis bergquistae* in Indonesia, only manadodioxan E (**127**) displayed antimicrobial activities against bacteria, namely *E. coli* and *B. cereus*. The presence of a carbonyl group at C-13 position in compound **126** possibly has sequestered the antimicrobial activity. However, both compounds were found to be inactive against *C. albicans*, and *S. cerevisiae* [77]. Using antibacterial bioassay-guided fractionation, two *O*-heterocyclic compounds belonging to pyranyl benzoate analogues of polyketide origin 2-(7-(2-Ethylbutyl)-2,3,4,4*a*,6,7-hexahydro-2-oxopyrano-[3,2*b*]-pyran-3-yl)-ethyl benzoate (**128**) and 2-(4*Z*)-2-ethyl-octahydro-6-oxo-3-(*E*)-pent-3-enylidene-pyrano-[3,2*b*]-pyran-7-yl-ethyl benzoate (**129**), were isolated from the ethyl acetate extract of *B. subtilis* MTCC 10407. Two additional homologs, i.e., (3-(methoxycarbonyl)-4-(5-(2-ethylbutyl)-5,6-dihydro-3-methyl-2*H*-pyran-2-yl)-butylbenzoate) (**130**) and [2-(8-butyl-3-ethyl-3,4,4*a*,5,6,8*a*-hexahydro-2*H*-chromen-6-yl)-ethylbenzoate] (**131**), were also isolated from the ethyl acetate extract from the host seaweed *Sargassum myriocystum*. Although compounds **130** and **131** displayed weaker antibacterial activities than compounds **128** and **129** these four compounds possessed similar structures suggesting the ecological and metabolic symbiosis between seaweeds and bacteria. It was evident from the study that the presence of dihydro-methyl-2*H*-pyran-2-yl propanoate system was essential to impart the antibacterial activity. Tetrahydropyran-2-one moiety of the tetrahydropyrano-[3,2*b*]-pyran-2(3*H*)-one system of compound **128** might be cleaved by the metabolic pool of seaweeds to afford biologically active methyl 3-(dihydro-3-methyl-2*H*-pyranyl)-propanoate moiety of compound **130** (which was shown to have no significant antibacterial activity in intact form) [78].

Fradimycins A (**132**) and B (**133**) isolated from marine *Streptomyces fradiae* strain PTZ0025 displayed antimicrobial activity against *S. aureus* [79]. Macrolactins, polyene cyclic macrolactones possess a wide range of biological activities including antimicrobial, antiviral and anticancer. The majority of macrolactins are produced by *Bacillus* sp., whilst glycosylated macrolactins A1 (**134**) and B1 (**135**) are frequently isolated from marine *Streptomyces* species. The position of the hydroxyl group (OH) or the introduction of a keto group (C=O) in the macrolactone ring affects the antimicrobial activity of macrolactins, while the introduction of ester groups at the C-7 in macrolactins leads to better antibacterial activity. Compounds **134** and **135** were less active than their corresponding ether-containing macrolactins, probably due to the attachment of a sugar moiety at C-7 position of both compounds. However, glycosylated macrolactins are reported to have better polar solubility in comparison to the non-glycosylated macrolactins [61]. From the culture broth of a marine *Bacillus* sp., compounds including macrolactones (Macrolactin X–Z, **136**–**138**) and macrolactinic acid (**139**) were isolated. These metabolites displayed potential antimicrobial activity against microbial strains, *B. subtilis* (KCTC 1021), *E. coli* (KCTC 1923), and *S. cerevisiae* (KCTC 7913). Macrolactins exhibit their antibacterial activity by inhibiting peptide deformylase in a dose-dependent manner. The position of hydroxy groups in the macrolactone ring is also important for antimicrobial activity of macrolactins. The hydroxy group at C-15 in macrolactone ring increases the antimicrobial activity of macrolactins, whereas introduction of carbonyl group at C-15 decreases antimicrobial activity. The number of ring members and the presence of a hydroxy group at C-7 and C-9 position have no effect on the antibacterial activity [62].

Chemical analysis of a marine-derived *Streptomyces* sp. (CMB-M0392) isolated from sediment collected off Heron Island, Queensland, Australia, yielded the benzothiazine ansamycins, heronamycin A (**140**) and herbimycin A (**141**). Compound **140** showed antimicrobial activity against *B. subtilis* ATCC 6051 and 6633, but no activity against *S. aureus* ATCC 25923 and 9144, *E. coli* ATCC 11775, *P. aeruginosa* ATCC 10145 or *C. albicans* ATCC 90028 while compound **141** showed antimicrobial activity against *B. subtilis* ATCC 6051 and 6633 and *C. albicans* ATCC 90028 [80]. Antibacterial polyketide compounds were isolated from the heterotrophic bacterium, *Bacillus amyloliquefaciens* which is associated with the edible red seaweed *Laurenciae papillosa*. Bioactivity-guided techniques resulted in the isolation of 3-(octahydro-9-isopropyl-2*H*-benzo[*h*]chromen-4-yl)-2-methylpropyl benzoate (**142**) and methyl 8-(2-(benzoyloxy)-ethyl)-hexahydro-4-(*E*)-pent-2-enyl)-2*H*-chromene-6-carboxylate (**143**), compounds of polyketide origin which demonstrated activity against human opportunistic food pathogenic microbes. Compounds **142** and **143** demonstrated significant antibacterial activity (inhibitory zone diameter of greater than 18 mm against *V. vulnificus*, 25 µg on disk) against these pathogenic bacteria and lesser activity against *A. hydrophilla* (14–16 mm, 25 µg on disk) and *V. parahemolyticus* ATCC17802TM (inhibitory zone diameter of 12–14 mm, 25 µg on disk). In general antibacterial activities of compound **142** were greater than **143**. Various molecular descriptor variables, such as bulk, polarizability (electronic), and lipophilicity (octanol/water partition coefficient) were found to significantly influence the antibacterial activities of these compounds. Although there was no significant dissimilarities in polarizability of compounds **142** and **143**, the activity of the latter was lesser (inhibition zone diameter, 16 mm against *V. vulnificus*; 25 µg on disk) than that of the former (18 mm against *V. vulnificus*; 25 µg on disk) due to the lesser lipophilicity associated with **143** compared to **142**. The higher lipophilicity of compound **142** enabled the compound to effectively penetrate the intermembrane lipoprotein barrier to enter the receptor location, resulting in greater bioactivity against food pathogenic bacteria [81]. 3-*O*-(*α*-d-ribofuranosyl)questin (**144**) and eurorubrin (**145**) isolated from the fungal strain *Eurotium cristatum* EN-220, an endophyte obtained from the marine alga *Sargassum thunbergii* displayed antimicrobial activity against *E. coli* but did not inhibit the growth of *S. aureus*, *Physalospora obtuse*, *A. brassicae*, *Valsa mali*, *A. solania*, and *Sclerotinia miyabeana* [82].

## 10. Isocoumarins

**Figure 8:**
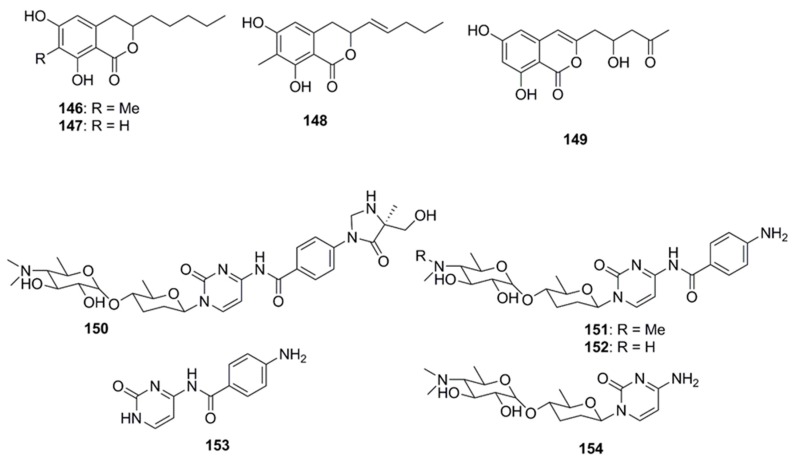
Antimicrobial isocoumarins (**146**–**149**) and nucleosides (**150**–**154**).

Antimicrobial dihydroisocoumarin derivatives penicisimpins A–C (**146**–**148**) were reported from a rhizosphere-derived fungus, *Penicillium simplicissimum* MA-332 obtained from a marine mangrove plant *Bruguiera sexangula* var. rhynchopetala (Figure 8). These isocoumarins possess a broad-spectrum of antibacterial and antifungal activities. Among these three isocoumarins, penicisimpins A (**146**) exhibited the greatest activity against *E. coli*, *P. aeruginosa*, *V. parahaemolyticus*, *V. harveyi*, and *C. gloeosprioides* (each with MIC value of 4 µg/mL) while the other two congeners (**147** and **148**) were only moderately active against these pathogens. It was determined that the methyl group attached at C-7 was responsible for the enhanced activity observed in compound **146** relative to **147**, while the double bond at C-11 was responsible for the decreased activity (compound **146** vs. **148**) [83]. The marine-derived fungal species *Penicillium vinaceum* has been reported to produce citreoisocoumarin (**149**) that displayed activity against *S. aureus* (19 mm inhibition zone) [47].

## 11. Nucleosides

Rocheicoside A (**150**), a nucleoside analogue possessing a novel 5-(hydroxymethyl)-5-methylimidazolidin-4-one substructure, and several other nucleosides such as plicacetin, norplicacetin, *p*-aminobenzanido uracil and cytosamine (**151**–**154**, Figure 8) have been isolated from the marine-derived actinomycete *Streptomyces rochei* 06CM016. These nucleosides have been described as potent antibiotics against microorganisms, including archaea, bacteria and eukarya. Rocheicoside A (**150**) displayed potential antimicrobial activity with MIC 4–16 µg/mL against a number of pathogens including *E. coli*, MRSA and *C. albicans* [84].

## 12. Miscellaneous Compounds

**Figure 9:**
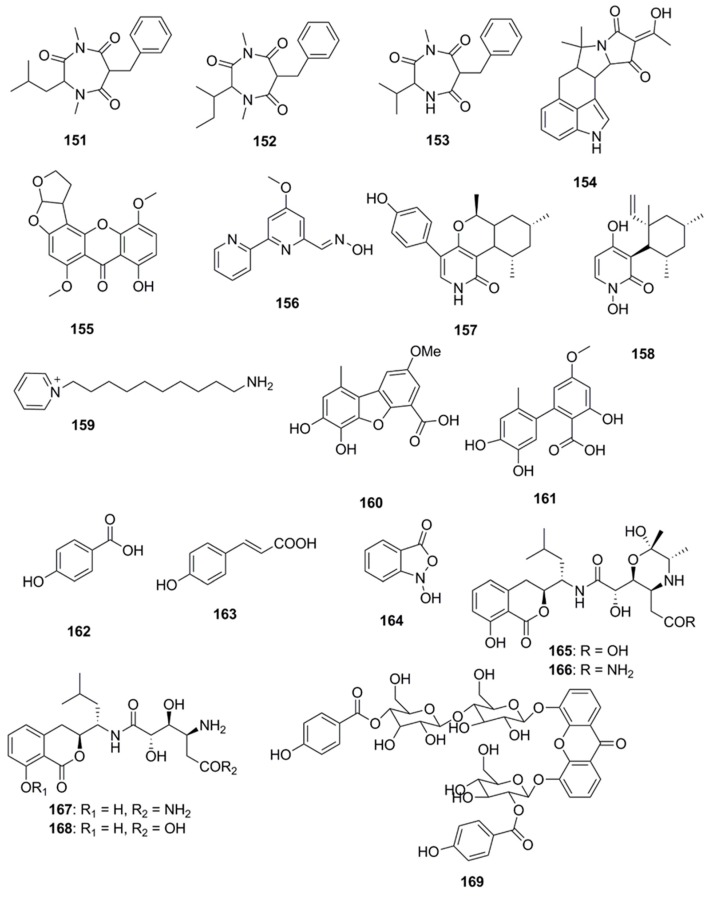
Antimicrobial miscellaneous compounds.

Several compounds which have not been classified under any of the above subheadings are discussed in this section and their chemical structures are illustrated in Figure 9. Terretrione A (**151**), terretrione C (**152**) and terretrione D (**153**) containing a 1,4-diazepane skeleton were isolated from an organic extract of the fungus *Penicillium* sp. CYE-87 derived from a tunicate-*Didemnum* sp. collected from the Suez Canal in Egypt. These compounds displayed antimicrobial activity against *C. albicans* but were inactive against *S*. *aureus* and *E. coli* [47,85]. Marine-derived fungi, *Penicillium vinaceum* species when investigated for antimicrobial compounds led to discovery of α-cyclopiazonic acid (**154**), which was active only against *E. coli* with an inhibition zone of 20 mm [47]. 5-methoxydihydrosterigmatocystin (**155**), a compound isolated from the marine-derived fungus, *Aspergillus versicolor* MF359, isolated from a marine sponge of *Hymeniacidon perleve* was active against *S. aureus* and *B. subtilis.* The compound did not display activity against MRSA and *P. aeruginosa* as the MIC was found to be >100 µg/mL against both the organisms [86]. Caerulomycin A (**156**), an antifungal compound isolated from marine actinomycetes *Actinoalloateichus cyanogriseus* showed potent activity against *Candida* isolates, *C. albicans* and *C. albicans* CO9, and two fluconazole resistant strains namely, *C. glabrata* HO5Fl and *C. krusei* GO3. The MICs of Caerulomycin A was in the range of 0.39–1.26 µg/mL. Furthermore, the MIC values obtained for Caerulomycin A against fluconazole resistant *C. glabrata* were comparable with the MIC values obtained for Amphotericin B [87]. Pyridones, trichodin A (**157**) and pyridoxatin (**158**), were extracted from both the mycelia and the culture broth of the marine fungus, *Trichoderma* sp. strain MF106 obtained from the Greenland Seas. Compounds **157** and **158** possess moderate antibiotic activities against the Gram-positive *B. subtilis*, *S. epidermidis*, MRSA and yeast, *C. albicans* but were inactive against *Trichophyton rubrum* [88]. A pyridinium compound, 1-(10-Aminodecyl) Pyridinium (**159**), isolated from the marine actinomycete, *Amycolatopsis alba* var. nov. DVR D4 demonstrated antimicrobial activities against Gram-positive and Gram-negative bacteria with MICs in the range of 70–160 µg/mL [89]. A dibenzofuran derivative porric acid D (**160**) and altenusin (**161**) were isolated from the methanol extract of the marine derived fungus, *Alternaria* sp., isolated from the Bohai Sea. These compounds were reported to have antibacterial activity against *S. aureus* [90]. Several small molecules including *p*-hydroxybenzoic acid (**162**), *trans*-cinnamic acid (**163**) and *N*-hydroxybenzoisoxazolone (**164**) were isolated from *Pseudoalteromonas flavipulchra* and showed antibacterial activity against *Vibrio anguillarum*. Greater growth inhibition was observed in *V. anguillarum* when a mixture of all three compounds was used compared to when each of the compounds was used individually, suggesting that they may act in a synergistic manner [72]. The antimicrobial compounds, bacilosarcin B (**165**) and C (**166**) and amicoumacin A (**167**) and B (**168**), were isolated from the culture broth of a marine-derived bacterium *B. subtilis*. The C-12 amide group of amicoumacin was found to be crucial for antibacterial activity against MRSA based on structural comparisons of amicoumacin A (**167**) and amicoumacin B (**168**). It was observed that only the compound amicoumacin A (**167**) with C-12′ amide groups exhibited antibacterial activities against *B. subtilis*, *S. aureus* and *L. hongkongensis*, which strongly supported the idea that the C-12′ amide group of amicoumcin acts as a pharmacophore in antibacterial activities. This conclusion was further supported by comparing antibacterial activities between compounds **167**/**168** and **165**/**166**. Compound **167** exhibited antibacterial activities against *B. subtilis*, *S. aureus* and *L. hongkongensis*, which were about six-fold higher than those of **168** [91]. Microluside A [4-(19-*p*-hydroxybenzoyloxy-*O*-*β*-d-cellobiosyl)-5-(30-*p*-hydroxybenzoyloxy-*O*-*β*-d-glucopyranosyl)xanthone] (**169**) is a unique *O*-glycosylated disubstituted xanthone isolated from the broth culture of *Micrococcus* sp. EG45 isolated from the Red Sea sponge *Spheciospongia vagabunda*. Compound **169** exhibited antibacterial activities against *E. faecalis* JH212, and *S. aureus* NCTC 8325 [92].

## 13. Synthetic Interventions

**Figure 10:**
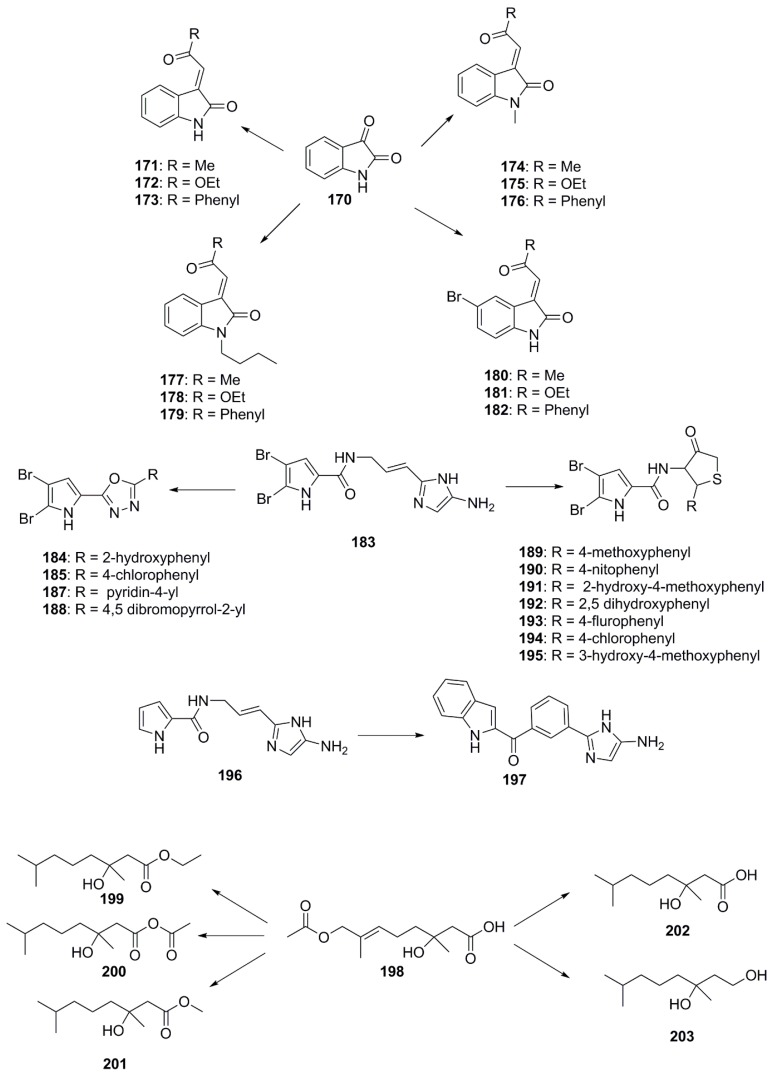
Semi-synthetic derivatives of MNPs.

Although naturally occurring marine natural products are bestowed with interesting structural features (both chemical and stereochemical), some pharmacophoric modifications are still required to improve their biological efficacy [93]. In this respect, several synthetic chemists are engaged in tailoring these natural products to obtain new chemical entities by modifying their natural structures [94] (Figure 10). Recently, Sakata et al. identified isatin (**170**), an algicidal substance produced by the marine bacterium *Pseudomonas* sp. C55a-2 isolated from coastal sea water of Kagoshima Bay in Japan and targeted this compound for synthetic modification. This particular compound was chosen as many strains belonging to the genera *Pseudomonas*, *Alteromonas* and *Pseudoalteromonas* sp. have previously been reported to use chemical defences in the form of extracellular agents like isatin. With background knowledge of the antifungal activities of isatin, several structural modifications were made to the compound including bromination of the C-5 carbon of the isatin ring, altering the length of its alkyl chain and *N*-protections, resulting in the development of molecules with potentially better pharmacophoric features. Structure-activity relationships revealed that a bromine substitution at the C-5 carbon atom in isatin derivatives led to a decrease in antibacterial activity when compared structurally to the parent isatin molecule. The antibacterial activity remains unchanged for *N*-methyl and *N*-butyl isatin derivatives, however, the addition of a free NH group to the structure of these compounds results in a decrease in antibacterial activity. Hence, it can be deduced that the free NH moiety of isatin is necessary for its potent inhibitory activities against fouling bacteria. The antibacterial activity was found to be better in the class of compounds wherein acetonyl moiety is introduced as functionality, compared to more extended hydrophobic benzoyl, as well as electron donating ethoxy group. Compounds **171**–**173** from this group exhibited a greater inhibitory effect compared to the parent compound **170** and to the *N*-protected isatins **174**–**179** and the 5-bromoisatin derivatives **180**–**182**. The presence of a 3-acetonylidene group and a free NH moiety in compounds **171**–**173** were determined to be crucial structural elements responsible for enhancing the antibacterial activity of these compounds [95].

Bromopyrrole alkaloids are produced by marine sponges and possess an array of diverse biological activities, including antimicrobial and antineoplastic activities [96,97,98]. Many of these molecules are readily identifiable by a 4,5-dibromopyrrole ring contained within their structure, with oroidin (**183**) being the best characterized of these alkaloids noted for its antibiofilm activity [99] In 2012, Rane et al. synthesized marine bromopyrrole alkaloid derivatives containing 1,3,4-oxadiazole and thiazolidinone and evaluated their antimicrobial and antibiofilm properties. Compounds **184**–**188** are among these synthetic oroidin derivatives containing 1,3,4-oxadiazole and have exhibited antimicrobial activity against representative Gram-positive and Gram-negative bacteria [100,101]. These compounds demonstrated antibacterial activity comparable to ciprofloxacin^®^ when used against *S. aureus* (MIC = 1.56 µg/mL). Further substitutions of 1-methyl-4,5-dibromopyrrole core with 4-thiazolidinone had been synthesized and tested for antibiofilm potentials against few Gram-positive bacteria. 4-thiazolidinone derivatives, compounds **189** and **190** showed antibiofilm activities (MIC = 0.78 µg/mL) 3-fold superior than those exerted following standard Vancomycin^®^ use (MIC = 3.125 µg/mL), while activity of compounds **191**–**194** was 2-fold (MIC = 1.56 µg/mL) higher against *S. aureus* biofilm formation. Compounds **189**–**195** showed equal antibiofilm activity against *S. epidermidis* compared to standard Vancomycin (MIC = 3.125 µg/mL) [102]. 

In 2014, Zidar et al. isolated marine alkaloids, clathrodin (**196**) and oroidin (**183**), from sponges of the genus, *Agelas*, which possess significant antimicrobial activity against the bacterial strains *E. faecalis*, *S. aureus* and *E. coli* and *C. albicans*. The research group synthesized several derivatives using oroidin as a template. The most bioactive of all these derivatives was found to be 4-phenyl-2-aminoimidazole (**197**), which exhibited an MIC_90_ value of 12.5 µM against Gram-positive bacteria and 50 µM against *E. coli* [103].

A new marine-derived monoterpenoid compound, penicimonoterpene (+)-1 (**198**), was isolated from *Penicillium chrysogenum* QEN-24S in 2014 and had shown antifungal activity against *A. brassicae* and potent antibacterial activity against marine bacteria *Aeromonas hydrophila*, *V. harveyi*, and *V. parahaemolyticus*. This activity pattern encouraged chemists to develop a number of derivatives of compound **198**. Modifications focused on variation of the substituents at the C-8 position, the carbon-carbon double bond at the C-6/7 position, and carboxyl substituents at the C-1 position. Compounds **199**–**203** were synthesized according to these modifications and found to be particularly active against *F. graminearum*. It was determined that oxidation of the methyl to a hydroxymethyl group at the C-8 position or replacement of the methyl ester group at C-1 by an ethyl ester significantly increased the antifungal activity of these compounds. Compounds with a reduced double bond at C-6/7 also showed better inhibitory activities against *gloeosporioides* and *F. graminearum* except for those containing an aldehyde group [104].

## 14. Genome Mining of Marine Microorganisms—The Future of Antimicrobial Discovery

Antimicrobial compound discovery has traditionally mainly relied on bioassay-guided approaches involving the cultivation of microorganisms under a variety of growth conditions, the subsequent screening of culture extracts for bioactivity and chemical characterization of the compounds produced. Over time however, this approach has led to the frequent re-isolation of known compounds, resulting in a drastic decline in research efforts being undertaken by research groups and pharmaceutical companies [105], causing a significant deficit in the number of novel, natural products available for commercial and medicinal use. In this age of antimicrobial resistance, the demand for functionally diverse, unique antimicrobials has never been greater. Fortunately, advances in sequencing and ‘-omics’ based technologies have revived the field of natural product discovery in recent years, owing in large part to the cost effectiveness and speed associated with next-generation sequencing. With more than 99,000 sequenced bacterial genomes currently publically available in the NCBI database [106] and sequence data from thousands of metagenome projects which can be accessed at the Genomes OnLine Database [107], researchers are now focusing their efforts on genome-guided investigations as a complementary approach to traditional bioactivity-guided methods in an effort to expedite the identification of ’talented’ microbes, which are likely to possess the biosynthetic machinery typically associated with antimicrobial compound production.

Biosynthetic gene clusters (BGCs) are specialised groups of genes located in close proximity to each other in bacterial genomes and encode successive steps in the biosynthesis of natural products. Sequence-based detection, analysis and functional elucidation of these clusters are paramount to unlocking the true biosynthetic potential which resides within a microorganism. However functional elucidation of the products of these BGCs is not always easy, since most are either poorly expressed or not expressed at all under common laboratory culture conditions. Such was the case observed for the model antibiotic-producing actinobacterium, *Streptomyces coelicolor*, which, prior to having its entire genome sequenced in 2002 [108] was thought to contain BGCs responsible for the production of six distinct metabolites, previously identified by classic molecular genetic approaches [109]. However, upon inspection of the organism’s complete genome sequence, 16 additional BGCs including BGCs potentially encoding nonribosomal peptide synthetases (NRPSs) and polyketide synthases (PKSs) were identified by bioinformatics-based predictions [110,111]. These clusters were deemed likely to encode enzymes involved in the synthesis of polyketides and nonribosomal peptides which are considered to be among the more valuable classes of microbial secondary metabolites from a biopharmaceutical perspective [112,113].

At the time of writing this review, data pertaining to over one million putative gene clusters currently resides in the Atlas of Biosynthetic gene Clusters which forms part of the Integrated Microbial Genomes component of the Joint Genome Institute (JGI IMG-ABC) [114]. With such a wealth of sequence information available, the challenges researchers now face primarily centre on how to effectively mine such a huge quantity of data in order to rapidly identify biosynthetically ’talented’ microorganisms and furthermore, how to prioritize which BGCs to investigate among such a vast collection of uncharacterized clusters when targeting a desired anti-microbial bioactivity. 

In this respect the Secondary Metabolite Bioinformatics Portal (SMBP), functions as a useful access point for investigators; containing website links to databases and tools used in genome mining and secondary metabolism research [115]. Automated tools such as antiSMASH [116] BAGEL [117] and PRISM [118] and databases such as Bactibase [119] ClusterMine360 [120] and MIBiG [121] represent just a few of the sophisticated in silico analytical tools which have quickly established themselves as the go-to resources for BGC identification, characterization and comparison. antiSMASH (antibiotics and Secondary Metabolite Analysis Shell) in particular is one of the most extensively used open-source BGC mining tools. Integrating and cross-linking several analysis tools including BLAST+, HMMer3 and FastTree, this computational platform not only facilitates the detection of known BGCs but can also detect unknown, BGC-like regions in genomes via ClusterFinder [122] an algorithm which uses Pfam domain, pattern based predictions to detect putative BGCs. ClusterFinder works on the premise that even the biosynthetic pathways for unknown compounds are likely to use the same broad families of enzymes for the catalysis of key reactions. The antiSMASH framework has been continuously developed [116,123] since its launch in 2010 [124]. antiSMASH version 4.0 was recently released in April 2017 [125] and contains improvements in prediction software for specialized secondary metabolites, including ribosomally synthesized and post-translationally modified peptides (RiPPs) and terpene products and has been expanded from BGC mining in bacteria and fungi to BGC mining in plants [126]. Another recently developed tool is EvoMining, which integrates evolutionary concepts related to the emergence of natural product biosynthesis into genome mining. It is a newly developed phylogenomics approach toward BGC analysis and has been successfully used in the retrieval of arseno-organic BGCs, which were previously unobtainable via antiSMASH analysis [127].

**Figure 11:**
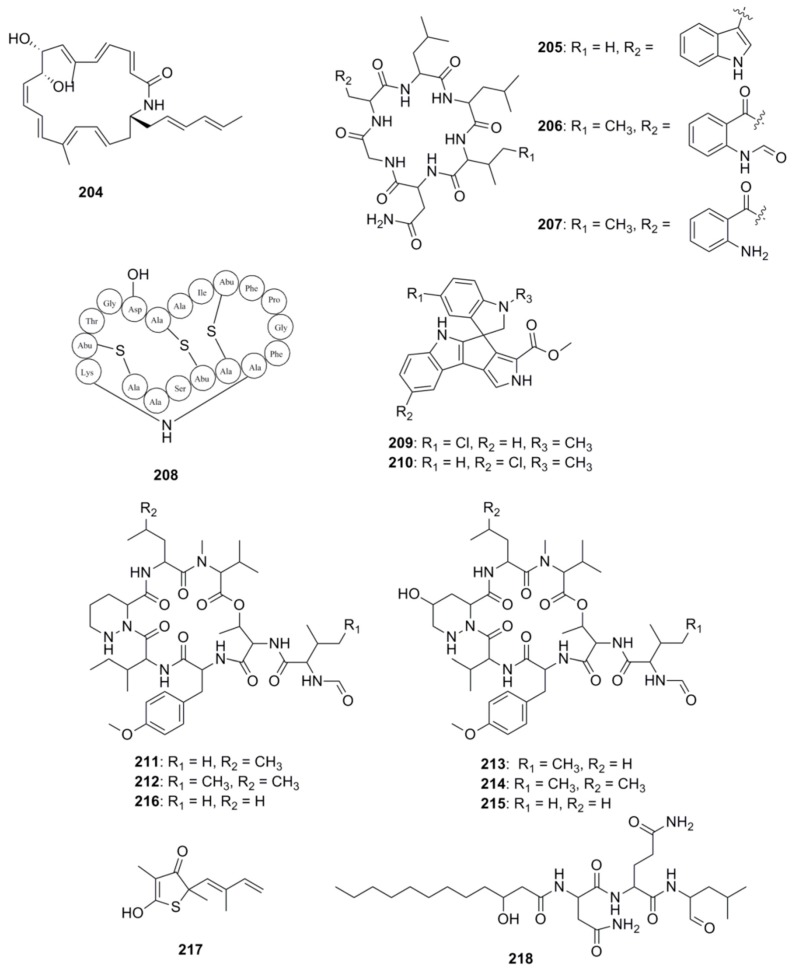
Antimicrobial compounds discovered via genome mining approaches.

With respect to the specific identification of antibacterial compounds and the pathways involved in their biosynthesis, genome mining approaches have proven useful in the identification of these types of compounds from marine derived bacteria. Figure 11 highlights a number of antimicrobial compounds discovered via genome mining approaches. The gene cluster involved in the biosynthesis of heronamide F (**204**) was identified following genome scanning of the deep-sea derived *Streptomyces* sp. SCSIO 03032. Heronamides are polyketide macrolactams which belong to a class of potent antifungal metabolites that are produced by marine-derived actinomycetes [128]. Confirmation of the involvement of this cluster in the biosynthesis of heronamide F was achieved by the functional confirmation of one of the genes in the cluster, namely *herO*, encoding a cytochrome P450 by gene knockout experiments [129]. Another example resides with three cyclohexapeptides, destomides B–D (**205**–**207**) which were initially isolated from the marine microbe *Streptomyces scopuliridis* SCSIO ZJ46. Destomide B was found to display antibacterial activity against strains of *Streptococcus pneumonia*, *Staphylococcus aureus* and methicillin-resistant *Staphylococcus epidermidis* (MRSE) [68]. Genome mining identified a putative 39-kb desotamide *dsa* gene cluster in the *S. scopuliridis* strain, and was determined to contain three NRPS genes. Subsequent heterologous expression of the *dsa* gene cluster in the heterologous host *S. coelicolor* M1152 confirmed its involvement of the biosynthesis of desotamides [130]. Finally, another recent example involving ribosomally synthesized and post-translationally modified peptides (RiPPs), centres on the new cinnamycin-like lantiobiotic, mathermycin (**208**) which has recently been isolated from the marine-derived strain, *Marinactinospora thermotolerans* SCSIO 00652 and possesses antimicrobial activities towards *Bacillus subtilis* [131]. Genome mining of the strain facilitated the identification of the BGC for mathermycin, while subsequent expression of the gene cluster in the heterologous *Streptomyces lividans* host system allowed its antibacterial activity to be assessed. 

Another popular approach in the identification of novel marine derived antimicrobials is to couple genomics with metabolomics, whereby genome mining together and metabolic profiling are used to identify novel natural products in marine microorganisms. Our group has previously employed this approach to identify bioactive compounds from *Streptomyces* sp. (SM8) isolated from the sponge *Haliclona simulans* which displayed antibacterial and antifungal activity [132]. Similarly, Paulus and co-workers employed both metabolomic and genomic profiling approaches on the marine *Streptomyces* sp. MP131-18 to identify a number of new biologically active compounds, including two new members of the bisindole pyrroles spirorindimycins, spiroindimicin E (**209**) and F (**210**). In addition, two new members of the α-pyrone lagunapyrone family, namely lagunapyrone D and E were also identified using similar approaches [133]. Another example of the successful use of this coupled approach resides in marformycins A-F (**211**–**216**), which display selective anti-microbial activity against *Micrococcus luteus*, *Propioniobacterium acnes* and *Propionobacterium granulosum*. The marformycin gene cluster was identified following genome scanning of the deep-sea sediment-derived *Streptomyces drozdowiczii* SCSIO 10141 strain. Confirmation of the involvement of this cluster in the biosynthesis of this group of cyclic peptides was achieved following in vivo inactivation studies coupled with metabolite identification [134].

Other techniques have also been developed to expedite the identification of BGCs, which include mining for the presence of self-resistance mechanisms within these gene clusters, allowing investigators to deduce the antibiotic compounds which an organism is likely to produce. Resistance mechanisms are characteristic traits associated with antibiotic producers, enabling an organism to avoid suicide from self-toxicity following the biosynthesis of its own molecule [135]. Resistance mechanisms include enzymes which degrade toxic compounds, efflux pumps for the effective removal of unwanted substances from the cell and target modification [136]. Wright and colleagues first used resistance as a discriminating criterion in 2013, demonstrating that organisms resistant to glycopeptide and ansamycin antibiotics are more likely to produce similar compounds [137]. Following this resistance based hypothesis increased the discovery rate of producers of the aforementioned antibacterial compounds by several orders of magnitude. Harnessing the success of this approach, Wright and colleagues further devised a method for isolating scaffold-specific antibacterial producers by taking advantage of the innate self-protection mechanisms employed by the producing organisms, isolating strains in the presence of a selective antibiotic [138]. In 2015, Moore and colleagues developed a target-directed genome mining method to identify BGCs [139]. As previously mentioned target modification is one of several resistance strategies employed by antibiotic producing bacteria and is effective in correlating an antibiotic to its mode of action. Since it is common for antibiotic producing bacteria to mutate and duplicate genes encoding proteins for resistance, Moore’s group reasoned that identifying target-duplicated genes which are co-clustered with BGCs would provide valuable information pertaining to the molecular targets of the BGC products without any prior knowledge of the molecule synthesized. Since antibiotic targets are often the product of housekeeping genes, Moore and colleagues screened 86 *Salinispora* bacterial genomes for duplicated copies of housekeeping genes and related them to their presence in BGCs. Using this approach they successfully identified a duplicated fatty acid synthase in the direct vicinity of an orphan hybrid PKS-NPRS gene cluster and prioritized this for investigation. Following cloning, heterologous expression and mutational analysis, the authors linked the gene cluster to the biosynthesis of thiolactomycin (**217**) a previously characterized fatty acid synthase inhibitor, and to the production of a group of unusual thiotetronic acid natural products [139]. In 2016, Oakley and colleagues provided experimental validation for target-directed genome mining in a fungal BGC system [140]. The group identified a proteasome subunit-encoding gene within a gene cluster in *Aspergillus nidulans* and hypothesized that the cluster may be responsible for the production of a proteasome inhibitor. Following a number of molecular genetic based strategies, the investigators determined that the product of the cluster was indeed a proteasome inhibitor, fellutamide B (**218**). Recently, Johnston and co-workers used resistance-based mining to predict natural products with new modes of action [141]. The authors used a retrobiosynthetic algorithm to mine biosynthetic scaffolds and resistance determinants to identify structures with unknown modes of action. Using this approach, the investigators determined that the telomycin family of natural products from *Streptomyces canus* possess a new antibacterial mode of action which targets cardiolipin, a bacterial phospholipid.

Tracanna and co-workers recently suggested another strategy as a means of prioritizing BGCs which may encode novel antibiotics, based on synergistic interactions [142]. Natural products can occur as synergistic pairs in nature, as in the case of cephamycin and clavulanic acid, two compounds which are naturally produced by *Streptomyces clavuligerus*. The BGCs for these compounds are intertwined in a ‘supercluster’ configuration [143,144]. This genetic conformation inspired the production of the antibiotic, Augmentin^®^, which is comprised of a combination of the β-lactam antibiotic, amoxicillin with the β-lactamase inhibitor, clavulanic acid. Synergistic pairs of natural products are particularly attractive commodities in the ongoing attempt to tackle antimicrobial resistance, as it is more difficult for a pathogen to develop resistance to both compounds. They suggest that thorough analysis surrounding the evolutionary history of the genes associated with large, hybrid BGCs may provide a valuable insight into whether these BGCs are in fact ‘superclusters’, responsible for the production of a number of different compounds which may be synergistic. 

It is important that in silico analytical tools keep pace with these genome-guided discovery strategies in order to expedite novel compound discovery. The Antibiotic Resistant Target Seeker (ARTS) is one such web tool which uses three criteria to detect known resistance genes as well as putative resistance house-keeping genes in actinobacterial genomes i.e., (i) duplication (ii) evidence for horizontal gene transfer (HGT) and (iii) localization within a BGC [145]. This inexpensive, computational analysis tool, facilitates high-throughput screening of bacterial genomes. Although the current focus of ARTS is on the analysis of actinobacterial genomes, the pipeline also works for other phyla and is being expanded to include reference sets for other taxa. Several other useful resources are available pertaining to the field of antimicrobial resistance, with the **C**omprehensive Antibiotic Resistance Database (CARD) [146] and Antibiotic Resistance Gene-ANNOTation (ARG-ANNOT) being the two most extensive databases [147].

## 15. Concluding Remarks and Future Prospects

The marine environment is home to a vast number of macro and microorganisms with untapped biosynthetic activities which are used to a large extent to ensure their survival in this diverse and often hostile habitat. This unique environment facilitates the biosynthesis of an array of secondary metabolites which act as chemical defenses and display a broad range of antimicrobial bioactivities. Nevertheless, despite extensive structural and stereo chemical diversity, only seven marine-derived metabolites have to date been approved as drugs, while 12 MNPs (or derivatives thereof) are currently in different phases of clinical trials.

As previously noted, none of the newly discovered marine natural products have as yet progressed to clinical trials, although a few of them are in preclinical studies. The slower pace of MNPs towards clinical trials is due to several factors that hinder their development as clinical agents. One of the major factors is the “continuous supply”. Large quantities of a compound are required to carry out biological assays to determine the site of action, specific targets, selectivity of the compound and its cytotoxicity. Irrespective of the potential applications of a functionally promising compound, a significant challenge faced by researchers is that several hundred grams of the compound are required for preclinical development, and multi kilogram quantities required for clinical trials. This is often one of the major bottle-necks in the development of MNP for clinical applications. Synthetic chemists around the world however are continuing to develop synthetic and semi-synthetic strategies to help overcome the supply issue surrounding MNPs, in an effort to satisfy the requirements to help bring these molecules to the preclinical stage and eventual development for use in the commercial or medicinal arenas.

MNPs can be chemically modified with various biosteric structural units to develop ‘drug-like’ molecules. Also, developments in mariculture (farming the growth of the organism in its natural environment) and aquaculture (culturing an organism under artificial conditions) have been attempted in order to solve the problem of sustainable supply of macroorganisms. However, the unique and sometimes exclusive conditions of the sea make cultivation or maintenance of isolated samples still very challenging and often impossible.

The preclinical pipeline also demands elaborate, mechanistic and pharmacokinetic studies to develop tailored MNPs, which in itself is a hugely challenging but nevertheless exciting task. Regardless of these challenges, the preclinical pipeline continues to supply studies with several hundred novel bioactive marine compounds with the potential for use as therapeutics. From a global perspective, the marine pharmaceutical pipeline remains very active, and now appears to have sufficient momentum to deliver additional antimicrobial compounds to the marketplace in the near future. The efficiency of various marine compounds against pathogens is very encouraging and there is no doubt that their exploitation and application will continue to develop**.** Genome mining has ushered in a renaissance in the field of natural product discovery, providing new hope in the ongoing search for novel antimicrobial compounds. This strategy allows researchers to harness the true biosynthetic potential which resides within diverse groups of marine microorganisms and offers an invaluable insight into not only the biosynthetic, but also the evolutionary and defensive strategies these organisms employ in the marine environment. Collaborative endeavours involving marine natural products chemistry with organic chemistry, medicinal chemistry, pharmacology, biology, bioinformatics and associated disciplines will help to ensure and facilitate an increase in marine natural product reaching the market as antimicrobial therapeutics.

## Figures and Tables

**Table 1:** Chemical classification of antimicrobial marine natural products (MNPs). MRSA: methicillin-resistant *Staphylococcus aureus*; MRSE: methicillin-resistant *Staphylococcus epidermidis* MSSA: methicillin-sensitive *Staphylococcus aureus*; MTCC: microbial type culture collection.

Compound	Source	Activity Against Pathogen
Alkaloids		
Pyranonigrin A (**16**)	*Penicillium brocae* MA-231	*S. aureus* (MIC 0.5 µg/mL)
		*V. harveyi* (MIC 0.5 µg/mL)
		*V. parahaemolyticus* (MIC 0.5 µg/mL)
		*A. brassicae* (MIC 0.5 µg/mL)
		*C. gloeosprioide* (MIC 0.5 µg/mL)
Pyranonigrin F (**17**)	*P. brocae* MA-231	*S. aureus* (MIC 0.5 µg/mL)
		*V. harveyi* (MIC 0.5 µg/mL)
		*Vibrio parahaemolyticus* (MIC 0.5 µg/mL*)*
		*Alternaria brassicae* (MIC 0.5 µg/mL)
		*C. gloeosprioide* (MIC 0.5 µg/mL)
Rubrumazine B (**18**)	*E. cristatum* EN-220	*Magnaporthe grisea* (MIC 64 µg/mL)
Echinulin (**19**)	*E. cristatum* EN-220	*S. aureus* (MIC 256 µg/mL)
Dehydroechinulin (**20**)	*E.cristatum* EN-220	*S. aureus* (MIC 256 µg/mL)
Variecolorin H (**21**)	*E. cristatum* EN-220	*S. aureus* (MIC 256 µg/mL)
Cristatumin A (**22**)	*E.cristatum*	*E. coli* (MIC 64 µg/mL)
		*S. aureus* (MIC 8 µg/mL)
Cristatumin D (**23**)	*E. cristatum*	*S. aureus* (Zone of inhibition 8 mm at 100 µg/disk)
Tardioxopiperazine A (**24**)	*E. cristatum*	*E. coli* (MIC 64 µg/mL)
		*S. aureus* (MIC 8 µg/mL)
Hemimycalin A (**27**)	*Hemimycale arabica*	*E. coli* (Inhibition zone 18 mm at 100 µg/disk)
		*C. albicans* (Inhibition zone 22 mm at 100 µg/disk)
Hemimycalin B (**28**)	*H. arabica*	*E. coli* (Inhibition zone 10 mm at 100 µg/disk)
		*C. albicans* (Inhibition zone 14 mm at 100 µg/disk)
(*Z*)-5-(4-hydroxybenzylidene)imidazolidine-2,4-dione (**29**)	*H. arabica*	*E. coli* (Inhibition zone 20 mm at 100 µg/disk)
		*C. albicans* (Inhibition zone 20 mm at 100 µg/disk)
Peniciadametizine A (**30**)	*Penicillium adametzioides*	*A. brassicae* (MIC 4 µg/mL)
Peniciadametizine B (**31**)	*P. adametzioides*	*A. brassicae* (MIC 32 µg/mL)
Penicibrocazine B (**33**)	*P. brocae*	*S. aureus* (MIC 32 µg/mL)
		*G.graminis* (MIC 0.25 µg/mL)
Penicibrocazine C (**34**)	*P. brocae*	*S. aureus* (MIC 0.25 µg/mL)
		*M. luteus* (MIC 0.25 µg/mL)
Penicibrocazine D (**35**)	*P. brocae*	*S. aureus* (MIC 8 µg/mL)
		*G.graminis* (MIC 8 µg/mL)
Penicibrocazine E (**36**)	*P. brocae*	*G. graminis* (MIC 0.25 µg/mL)
Crambescidin 800 (**37**)	*Clathria cervicornis*	*A. baumannii* (MIC 2 µg/mL)
		*K. pneumonia* (MIC 1 µg/mL)
		*P. aeruginosa*. (MIC 1 µg/mL)
Xinghaiamine A (**38**)	*Streptomyces xinghaiensis*	*S. aureus* (MIC 0.69 µM)
		*B. subtilis* (MIC 0.35 µM)
		*E. coli* (MIC 0.17 µM)
		*A. baumanii* (MIC 2.76 µM)
		*P. aeruginosa* (MIC 11 µM)
		MRSA 5301 (MIC 5.52 µM)
		MRSA 5438 (MIC 2.76 µM)
		MRSA 5885 (MIC 5.52 µM)
Hyrtioerectine D (**39**)	*Hyrtios* sp.	*C. albicans* (Zone of inhibition 17 mm at 100 µg/disk)
		*S. aureus* (Zone of inhibition 20 mm at 100 µg/disk)
		*P. aeruginosa* (Zone of inhibition 9 mm at 100 µg/disk)
Hyrtioerectine E (**40**)	*Hyrtios* sp.	*C. albicans* (Zone of inhibition 19 mm at 100 µg/disk)
		*S. aureus* (Zone of inhibition 10 mm at 100 µg/disk)
		*P. aeruginosa* (Zone of inhibition 9 mm at 100 µg/disk)
Hyrtioerectine F (**41**)	*Hyrtios* sp.	*C. albicans* (Zone of inhibition 14 mm at 100 µg/disk)
		*S. aureus* (Zone of inhibition 16 mm at 100 µg/disk)
		*P. aeruginosa* (Zone of inhibition 9 mm at 100 µg/disk)
Ageloxime B (**42**)	*Agelas mauritiana*	*C. neoformans* (MIC 5 µg/mL)
		*S. aureus* (MIC 7 µg/mL)
Ageloxime D (**43**)	*A. mauritiana*	*C. neoformans* (MIC 6 µg/mL)
Zamamidine D (**44**)	*Amphimedon* sp.	*E. coli* (MIC 32 µg/mL)
		*S. aureus* (MIC 8 µg/mL)
		*B. subtilis* (MIC 8 µg/mL)
		*M. luteus* (MIC 8 µg/mL)
		*A. niger* (MIC 16 µg/mL)
		*T. mentagrophytes* (MIC 8 µg/mL)
		*C. albicans* (MIC 16 µg/mL)
		*C. neoformans* (MIC 2 µg/mL)
Adametizine A (**45**)	*P. adametzioides* AS-53	*S.aureus* (MIC 8 µg/mL)
		*A. hydrophilia* (MIC 8 µg/mL)
		*V. harveyi* (MIC 32 µg/mL)
		*V. parahaemolyticus* (MIC 8 µg/mL)
		*G. graminis* (MIC 16 µg/mL)
Adametizine B (**46**)	*P.adametzioides* AS-53	*S. aureus* (MIC 64 µg/mL)
Iso-Agelasidine B (**47**)	*A. nakamurai*	*C. albicans* (MIC 2.34 µg/mL)
(−)-Agelasidine C (**48**)	*A. nakamurai*	*C. albicans* (MIC 2.34 µg/mL)
Iso-agelasine C (**49**)	*A. nakamurai*	*S. aureus* (MIC 75 µg/mL)
		*E. coli* (MIC 150 µg/mL)
		*P. vulgaris* (MIC 19 µg/mL)
		*C. albicans* (MIC 5 µg/mL)
Agelasine B (**50**)	*A. nakamurai*	*P. vulgaris* (MIC 19 µg/mL)
		*C. albicans* (MIC 2 µg/mL)
Agelasine J (**51**)	*A. nakamurai*	*S. aureus* (MIC 75 µg/mL)
		*E. coli* (MIC 75 µg/mL)
		*P. vulgaris* (MIC 9 µg/mL)
		*C. albicans* (MIC 0.6 µg/mL)
Nemoechine G (**52**)	*A. nakamurai*	*S. aureus* (MIC 150 µg/mL)
		*E. coli* (MIC 75 µg/mL)
		*P. vulgaris* (MIC 9 µg/mL)
		*C. albicans* (MIC 0.6 µg/mL)
Brevianamide F (**53**)	*Penicillium vinaceum*	*C. albicans* (Zone of inhibition 25 mm at 100 µg/disk)
		*S. aureus* (Zone of inhibition 19 mm at 100 µg/disk)
*N*-(2-hydroxyphenyl)-2-phenazinamine (**54**)	*Nocardia dassonvillei*	*C. albicans* (MIC of 64 µg/mL)
Terpenoids		
Puupehenol (**55**)	*Dactylospongia* sp.	*S. aureus* (Zone of inhibition 4 mm at 10 µg/disk)
		*B. cereus* (Zone of inhibition 4 mm at 10 µg/disk)
Puupehenone (**56**)	*Dactylospongia* sp.	*S. aureus* (Zone of inhibition 3 mm at 10 µg/disk)
		*B. cereus* (Zone of inhibition 3 mm at 10 µg/disk)
Penicibilaene A (**57**)	*Penicillium bilaiae*	*C. gloeosporioides* (MIC 1 µg/mL)
Penicibilaene B (**58**)	*P. bilaiae*	*C. gloeosporioides* (MIC 0.1 µg/mL)
Aspergillusene A (**59**)	*Aspergillus sydowii*	*K. pneumonia* (MIC 21 µM)
		*A. hydrophila* (MIC 4.3 µM)
(*Z*)-5-(Hydroxymenthyl)-2-(6′)-methylhept-2′-en-2′-yl)-phenol (**60**)	*A. sydowii*	*K. pneumonia* (MIC 11 µM)
Sydonic acid (**61**)	*A. sydowii*	*E. faecalis* (MIC 19 µM)
12-hydroxy isolaurene (**62**)	*Laurencia obtuse*	*B. subtilis* (MIC 46 µg/mL)
		*S. aureus* (MIC 52 µg/mL)
8,11-dihydro-12-hydroxy isolaurene (**63**)	*L. obtuse*	*C. albicans* (MIC 120 µg/mL)
		*A. fumigatus* (MIC 200 µg/mL)
		*B. subtilis* (MIC 39 µg/mL)
		*S. aureus* (MIC 31 µg/mL)
Isolauraldehyde (**64**)	*L. obtuse*	*C. albicans* (MIC 70 µg/mL)
		*A. fumigatus* (MIC 100 µg/mL)
		*B. subtilis* (MIC 35 µg/mL)
		*S. aureus* (MIC 27 µg/mL)
Napyradiomycin 1 (**65**)	*Streptomyces strain*	MRSA (MIC 16 µg/mL)
Napyradiomycin 2 (**66**)	*Streptomyces strain*	MRSA (MIC 64 µg/mL)
Napyradiomycin B2 (**67**)	*Streptomyces strain*	MRSA (MIC 32–64 µg/mL)
Napyradiomycin B3 (**68**)	*Streptomyces strain*	MRSA (MIC 2 µg/mL)
Napyradiomycin B4 (**69**)	*Streptomyces strain*	MRSA (MIC 32 µg/mL)
Dixiamycins A (**70**)	*Streptomyces* sp.	*E. coli* (MIC 8 µg/mL)
		*S. aureus* (MIC 8 µg/mL)
		*B. subtilis* (MIC 16 µg/mL)
		*B. thuringensis* (MIC 4 µg/mL)
Dixiamycins B (**71**)	*Streptomyces* sp.	*E. coli* (MIC 8 µg/mL)
		*S. aureus* (MIC 16 µg/mL)
		*B. subtilis* (MIC 16 µg/mL)
		*B. thuringensis* (MIC 8 µg/mL)
Oxiamycin (**72**)	*Streptomyces* sp.	*E. coli* (MIC 64 µg/mL)
		*S. aureus* (MIC 128 µg/mL)
Chloroxiamycin (**73**)	*Streptomyces* sp.	*E. coli* (MIC 64 µg/mL)
		*S. aureus* (MIC 64 µg/mL)
		*B. thuringensis* (MIC 64 µg/mL)
Xiamycin A (**74**)	*Streptomyces* sp.	*E. coli* (MIC 64 µg/mL)
		*S. aureus* (MIC 64 µg/mL)
Sarcotrocheliol acetate (**75**)	*Sarcophyton trocheliophorum*	*S. aureus* (MIC 1.7 µM)
		MRSA (MIC 1.7 µM)
		*Acinetobacter* pp*.* (MIC 4.3 µM)
Sarcotrocheliol (**76**)	*S. trocheliophorum*	*S. aureus* (MIC 1.5 µM)
		MRSA (MIC 3.0 µM)
		*Acinetobacter* sp. (MIC 3.0 µM)
Cembrene-C (**77**)	*S. trocheliophorum*	*C. albicans* (MIC 0.7 µM)
		*A. flavus* (MIC 0.7 µM)
Sarcophine (**78**)	*S. trocheliophorum*	*S. aureus* (MIC 9.4 µM)
		MRSA (MIC 9.4 µM)
		*Acinetobacter* sp. (MIC 9.4 µM)
Palustrol (**79**)	*S. trocheliophorum*	*S. aureus* (MIC 6.6 µM)
		MRSA (MIC 6.6 µM)
		*Acinetobacter* sp. (MIC 11.1 µM)
epi-Ilimaquinone (**80**)	*Hippospongia* sp.	MRSA (MIC 63 µg/mL)
		*S. aureus* (MIC 31 µg/mL)
		Vancomycin resistant *E. faecium* (MIC 16 µg/mL)
		Amphotericin-resistant *C. albicans* (MIC 125 µg/mL)
Penicillosides A (**81**)	*Penicillium* sp.	*C. albicans* (inhibition zone 23 mm at 100 µg/disk)
Penicillosides B (**82**)	*Penicillium* sp.	*S. aureus* (inhibition zone 19 mm at 100 µg/disk)
		*E. coli* (inhibition zone 20 mm at 100 µg/disk)
Ieodoglucomide A (**83**)	*Bacillus licheniformis*	*S. aureus* (MIC 8 µg/mL)
		*B. subtilis* (MIC 16 µg/mL)
		*B. cereus* (MIC 16 µg/mL)
		*S. typi* (MIC 16 µg/mL)
		*E. coli* (MIC 8 µg/mL)
		*P. aeruginosa* (MIC 8 µg/mL)
		*A. niger* (MIC 32 µg/mL)
		*C. albicans* (MIC 32 µg/mL)
Ieodoglucomide B (**84**)	*B. licheniformis*	*S. aureus* (MIC 8 µg/mL)
		*B. subtilis* (MIC 16µg/mL)
		*B. cereus* (MIC 8 µg/mL)
		*S. typi* (MIC 16 µg/mL)
		*E. coli* (MIC 16µg/mL)
		*P. aeruginosa* (MIC 8 µg/mL)
		*A. niger* (MIC 32 µg/mL)
		*C. albicans* (MIC 16 µg/mL)
Iedoglucomide C (**85**)	*Bacillus licheniformis*	*S. aureus* (MIC 0.03 µM)
		*B. subtilis* (MIC 0.03 µM)
		*B. cereus* (MIC 0.01 µM)
		*S. typi* (MIC 0.01 µM)
		*E. coli* (MIC 0.01 µM)
		*P. aeruginosa* (MIC 0.01 µM)
		*A. niger* (MIC 0.05 µM)
		*R. solani* (MIC 0.05 µM)
		*C. acutatum* (MIC 0.03 µM)
		*B. cenerea* (MIC 0.03 µM)
		*C. albicans* (MIC 0.03 µM)
Iedoglycoloipd (**86**)	*B. licheniformis*	*S. aureus* (MIC 0.03 µM)
		*B. subtilis* (MIC 0.05 µM)
		*B. cereus* (MIC 0.03 µM)
		*S. typi* (MIC 0.05 µM)
		*E. coli* (MIC 0.03 µM)
		*P. aeruginosa* (MIC 0.03 µM)
		*A. niger* (MIC 0.03 µM)
		*R. solani* (MIC 0.05 µM)
		*C. acutatum* (MIC 0.03 µM)
		*B. cenerea* (MIC 0.03 µM)
		*C. albicans* (MIC 0.05 µM)
Gageotetrin A (**87**)	*Bacillus subtilis*	*C. acutatum* (MIC 0.03 µM)
		*B. cinera.* (MIC 0.03 µM)
		*S. aureus* (MIC 0.03 µM)
		*B. subtilis* (MIC 0.03 µM)
Gageotetrin B (**88**)	*B. subtilis*	*C. acutatum* (MIC 0.01 µM)
		*B. cinera.* (MIC 0.01 µM)
		*S. aureus* (MIC 0.04 µM)
		*B. subtilis* (MIC 0.02 µM)
Gageotetrin C (**89**)	*B. subtilis*	*C. acutatum* (MIC 0.02 µM)
		*B. cinera* (MIC 0.01 µM)
		*S. aureus* (MIC 0.04 µM)
		*B. subtilis* (MIC 0.04 µM)
Lauramide diethanolamine (**90**)	*Streptomyces* sp.	*B. subtilis* (MIC 0.055 µg/mL)
		*E. coli* (MIC 0.055 µg/mL)
		*P. aeruginosa* (MIC 0.011 µg/mL)
		*S. aureus* (MIC 0.011 µg/mL)
		*S. cerevisiae* (MIC 0.022 µg/mL)
Linieodolide A (**91**)	*Bacillus* sp.	*B. subtilis* (MIC 8 µg/mL)
		*E. coli* (MIC 8 µg/mL)
		*S. cerevisiae* (MIC 32 µg/mL)
Linieodolide B (**92**)	*Bacillus* sp.	*B. subtilis* (MIC 64 µg/mL)
		*E. coli* (MIC 64 µg/mL)
		*S. cerevisiae* (MIC 128 µg/mL)
Dysiroid A (**93**)	*Dysidea* sp.	*S. aureus* ATCC 29213 (MIC 4 µg/mL)
		*S. aureus* ATCC 43300(MIC 8 µg/mL)
		*E. faecalis* ATCC 29212(MIC 4 µg/mL)
		*B. licheniformis* ATCC 10716 (MIC 16 µg/mL)
Dysiroid B (**94**)	*Dysidea* sp.	*S. aureus* ATCC 29213 (MIC 4 µg/mL)
		*S. aureus* ATCC 43300(MIC 4 µg/mL)
		*E. faecalis* ATCC 29212(MIC 4 µg/mL)
		*B. licheniformis* ATCC 10716 (MIC 8 µg/mL)
Halistanol sulfate A (**95**)	*Petromica ciocalyptoides*	*S. mutans* clinical isolate (MIC 15 µg/mL)
		*S. mutans* UA159 (MIC 15 µg/mL)
Peptides		
Rodriguesines A and B (**96** and **96a**)	*Didemnum* sp.	*S. mutans* clinical isolate (MIC 31 µg/mL)
		S. mutans UA159 (MIC 62 µg/mL)
Cyclo-(l-valyl-d-proline) (**97**)	*Rheinheimera japonica*	*S. aureus* (Zone of inhibition 17 mm at 0.5 mg/mL)
		*V. parahaemolyticus* (MIC 0.05 µg/mL)
		*V. vulnificus* (MIC 5 µg/mL)
		*M. luteus* (MIC 5 µg/mL)
Cyclo-(l-phenylalanyl-d-proline (**98**)	*R. japonica*	*S. aureus* (Zone of inhibition 15 mm at 0.5 mg/mL)
Cyclo-(S-Pro-R-Leu) (**99**)	*Haliclona oculata*	*V. parahaemolyticus* (MIC 0.5 µg/mL)
		*V. vulnificus* (MIC 5 µg/mL)
		*B. cereus* (MIC 0.05 µg/mL)
Isaridin G (**100**)	*Beauveria felina EN-135*	*E. coli* (MIC 64 µg/mL)
Desmethylisaridin G (**101**)	*B. felina EN-135*	*E. coli* (MIC 64 µg/mL)
Desmethylisaridin C1(**102**)	*B. felina EN-135*	*E. coli* (MIC 8 µg/mL)
Isaridin E (**103**)	*B. felina EN-135*	*E. coli* (MIC 16 µg/mL)
Desotamide (**104**)	*Streptomyces scopuliridis SCSIO ZJ46*	*S. pnuemoniae* (MIC 13 µg/mL)
		*S. aureus* (MIC 16 µg/mL)
		MRSE (MIC 32 µg/mL)
Desotamide B (**105**)	*S. scopuliridis SCSIO ZJ46*	*S. pnuemoniae* (MIC 13 µg/mL)
		*S. aureus* (MIC 16 µg/mL)
		MRSE (MIC 32 µg/mL)
Halogenated Compounds		
2-(2′,4′-dibromophenoxy)-3,5-dibromophenol (**106**)	*Dysidea granulosa*	MSSA (MIC 1 mg/L)
		*L. monocytogenes* (MIC 2 mg/L)
		*B. cereus* (MIC 0.1 mg/L)
		*C. diffiile* (MIC 4 mg/L)
		MRSA (MIC 0.1 mg/L)
		*Salmonella* sp. (MIC 1 mg/L)
		*E. coli* O157:H7 (MIC 8 mg/L)
		*Pseudomonas* (MIC 4 mg/L)
		*K. pneumoniae* (MIC 0.1 mg/L)
		*N. gonorrhoeae* (MIC 2 mg/L)
		*A. baumannii* (MIC 16 mg/L)
		*C. jejuni* (MIC 5 mg/L)
2-(2′,4′-dibromophenoxy)-3,4,5-tribromophenol (**107**)	*Dysidea granulosa*	*L. monocytogenes* (MIC 0.1 mg/L)
		*C. diffiile* (MIC 10 mg/L)
		MRSA (MIC 0.1 mg/L)
		*Salmonella* sp. (MIC 10 mg/L)
		*C. jejuni* (MIC 5 mg/L)
2-(2′,4′-dibromophenoxy)-4,6-dibromophenol (**108**)	*Dysidea* sp.	*L. monocytogenes* (MIC 1 mg/L)
		*B. cereus* (MIC 5 mg/L)
		*S. pneumoniae* (MIC 5 mg/L)
		*C. diffiile* (MIC 10 mg/L)
		MRSA (MIC 1 mg/L)
		*Salmonella* sp. (MIC 10 mg/L)
		*E. coli* O157:H7 (MIC 10 mg/L)
		*K. pneumoniae* (MIC 5 mg/L)
		*C. jejuni* (MIC 5 mg/L)
Aplysamine 8 (**109**)	*Pseudoceratina purpurea*	*E. coli* (MIC 125 µg/mL)
		*S. aureus* (MIC 31 µg/mL)
Sphaerodactylomelol (**113**)	*Sphaerococcus coronopifolius*	*S. aureus* (MIC 96 μM)
Bromosphaerol (**114**)	*Sphaerococcus coronopifolius*	*S. aureus* (MIC 22 µM)
12*R*-hydroxybromosphaerol (**115**)	*Sphaerococcus coronopifolius*	*S. aureus* (MIC 6 μM)
6-bromoindolyl-3-acetic acid (**116**)	*Pseudoalteromonas flavipulchra*	*V. anguillarum* (MIC 0.25 mg/mL)
Nagelamide X (**117**)	*Agelas* sp.	*S. aureus* (MIC 8 μg/mL)
		*M. luteus* (MIC 8 µg/mL)
		*A. niger* (IC_50_ 32 µg/mL)
		*T. mentagrophytes* (IC_50_ 16 µg/mL)
		*C. albicans* (IC_50_ 2 µg/mL)
Nagelamide Y (**118**)	*Agelas* sp.	*C. albicans* (IC_50_ 2 µg/mL)
Nagelamide Z (**119**)	*Agelas* sp.	*S. aureus* (MIC 16 µg/mL)
		*M. luteus* (MIC 8 µg/mL)
		*A. niger* (IC_50_ 4 µg/mL)
		*T. mentagrophytes* (IC_50_ 4 µg/mL)
		*C. albicans* (IC_50_ 0.25 µg/mL)
		*C. neoformans* (IC_50_ 2 µg/mL)
Ianthelliformisamine A (**120**)	*Suberea ianthelliformis*	*P. aeruginosa* (IC_50_ 7 µM)
Ianthelliformisamine C (**122**)	*Suberea ianthelliformis*	*P. aeruginosa* (IC_50_ 9 µM)
		*S. aureus* (IC_50_ 4µM)
Polyketides		
*N*-acetyl-*N*-demethylmayamycin (**123**)	*Streptomyces* sp. *182SMLY*	MRSA (MIC 20 µM)
Lindgomycin (**124**)	*Lindgomycetaceae strain*	*B. subtilis* (MIC 2.2 µM)
		*X. campestris* (MIC 17.8 µM)
		*S. epidermidis* (MIC 4.6 µM)
		*S. aureus* (MIC 2.7 µM)
		*S. aureus* (MRSA) (MIC 5.1 µM)
		*C. albicans* (MIC 5.7 µM)
		*S. tritici* (MIC 5.1 µM)
		*P. acnes* (MIC 4.7 µM)
Ascosetin (**125**)	*Lindgomycetaceae strain*	*B. subtilis* (MIC 3.4 µM)
		*X. campestris* (MIC 14.8 µM)
		*S. epidermidis* (MIC 6.3 µM)
		*S. aureus* (MIC 2.9 µM)
		*S. aureus* (MRSA) (MIC 3.2 µM)
		*C. albicans* (MIC 8 µM)
		*S. tritici* (MIC 10 µM)
		*P. acnes* (MIC 2.8 µM)
Manadodioxan E (**127**)	*Plakortis bergquistae*	*E. coli* (Zone of inhibition 16 mm at 10 µg/disk)
		*B. cereus* (Zone of inhibition 9 mm at 10 µg/disk)
2-(7-(2-Ethylbutyl)-2,3,4,4*a*,6,7-hexahydro-2-oxopyrano-[3,2*b*]-pyran-3-yl)-ethyl benzoate (**128**)	*B. subtilis* MTCC 10407	*V. parahemolyticus* (Zone of inhibition 11 mm at 10 µg/disk)
		*V. vulnificus* (Zone of inhibition 13 mm at 10 µg/disk)
		*A. hydrophilla* (Zone of inhibition 18 mm at 10 µg/disk)
2-((4*Z*)-2-ethyl-octahydro-6-oxo-3-((*E*)-pent-3-enylidene)-pyrano-[3,2*b*]-pyran-7-yl)-ethyl benzoate (**129**)	*B. subtilis* MTCC 10407	*V. parahemolyticus* (Zone of inhibition 11 mm at 10 µg/disk/mL)
		*V. vulnificus* (Zone of inhibition 14 mm at 10 µg/disk)
		*A. hydrophilla* (Zone of inhibition 15 mm at 10 µg/disk)
3-(methoxycarbonyl)-4-(5-(2-ethylbutyl)-5,6-dihydro-3-methyl-2*H*-pyran-2-yl)-butyl benzoate (**130**)	*Sargassum myriocystum*	*V. parahemolyticus* (Zone of inhibition 7 mm at 10 µg/disk)
		*V. vulnificus* (Zone of inhibition 7 mm at 10 µg/disk)
		*A. hydrophilla* (Zone of inhibition 8 mm at 10 µg/disk)
2-(8-butyl-3-ethyl-3,4,4*a*,5,6,8*a*hexahydro-2*H*-chromen-6-yl)-ethyl benzoate (**131**)	*Sargassum myriocystum*	*V. parahemolyticus* (Zone of inhibition 9 mm at 10 µg/disk)
		*V. vulnificus* (Zone of inhibition 8 mm at 10 µg/disk
		*A. hydrophilla* (Zone of inhibition 7 mm at 10 µg/disk)
Fradimycin A (**132**)	*Streptomyces fradiae*	*S. aureus* (MIC 6 µg/mL)
Fradimycin B (**133**)	*Streptomyces fradiae*	*S. aureus* (MIC 2 µg/mL)
Glycosylated Macrolactin A1 (**134**)	*Streptomyces* sp.	*B. subtilis* (MIC 0.027 µg/mL)
		*E. coli* (MIC 0.022 µg/mL)
		*P. aeruginosa* (MIC 0.055 µg/mL)
		*S. aureus* (MIC 0.055 µg/mL)
		*S. cerevisiae* (MIC 0.022 µg/mL)
Glycosylated Macrolactin B1 (**135**)	*Streptomyces* sp.	*B. subtilis* (MIC 0.055 µg/mL)
		*E. coli* (MIC 0.022 µg/mL)
		*P. aeruginosa* (MIC 0.055 µg/mL)
		*S. aureus* (MIC 0.055 µg/mL)
		*S. cerevisiae* (MIC 0.022 µg/mL)
Macrolactin X (**136**)	*Bacillus* sp.	*B. subtilis* (MIC 16 µg/mL)
		*E. coli* (MIC 16 µg/mL)
		*S. cerevisiae* (MIC 64 µg/mL)
Macrolactin Y (**137**)	*Bacillus* sp.	*B. subtilis* (MIC 16 µg/mL)
		*E. coli* (MIC 32 µg/mL)
		*S. cerevisiae* (MIC 64 µg/mL)
Macrolactin Z (**138**)	*Bacillus* sp.	*B. subtilis* (MIC 16 µg/mL)
		*E. coli* (MIC 32 µg/mL)
		*S. cerevisiae* (MIC 64 µg/mL)
Macrolactinic acid (**139**)	*Bacillus* sp.	*B. subtilis* (MIC 64 µg/mL)
		*E. coli* (MIC 32 µg/mL)
		*S. cerevisiae* (MIC 128 µg/mL)
Heronamycin A (**140**)	*Streptomyces* sp.	*B. subtilis* ATCC 6051 (MIC 18 µM)
		*B. subtilis* ATCC (MIC 14 µM)
Herbimycin A (**141**)	*Streptomyces* sp.	*B. subtilis* ATCC 6051 (MIC 5 µM)
		*B. subtilis* ATCC (MIC 5 µM)
		*C. albicans* ATCC 90028 (MIC 7 µM)
3-(Octahydro-9-isopropyl-2*H*-benzo[h]chromen-4-yl)-2-methylpropyl benzoate (**142**)	*Bacillus amyloliquefaciens*	*V. parahemolyticus* (Zone of inhibition 14 mm at 25 µg/disk)
		*V. vulnificus* (Zone of inhibition 16 mm at 25 µg/disk)
		*A. hydrophilla* (Zone of inhibition 18 mm at 25 µg/disk)
Methyl 8-(2-(benzoyloxy)ethyl)-hexahydro-4-((*E*)-pent-2-enyl)-2*H*-chromene-6-carboxylate (**143**)	*Bacillus amyloliquefaciens*	*V. parahemolyticus* (Zone of inhibition 12 mm at 25 µg/disk)
		*V. vulnificus* (Zone of inhibition 14 mm at 25 µg/disk)
		*A. hydrophilla* (Zone of inhibition 16 mm at 25 µg/disk)
3-*O*-(α-d-ribofuranosyl)questin (**144**)	*Eurotium cristatum*	*E. coli* (MIC 32 µg/mL)
Eurorubrin (**145**)	*Eurotium cristatum*	*E. coli* (MIC 64 µg/mL)
Isocoumarins		
Penicisimpins A (**146**)	*Penicillium simplicissimum MA-332*	*A. hydrophilia* (MIC > 64µg/mL)
		*E. coli* (MIC 4 µg/mL)
		*M. luteus* (MIC 8 µg/mL)
		*P. aeruginosa* (MIC 4 µg/mL)
		*V. alginolyticus* (MIC 8µg/mL)
		*V. harveyi* (MIC 4 µg/mL)
		*V. parahaemolyticus* (MIC 4 µg/mL)
		*C. gloeosprioides* (MIC 4 µg/mL)
Penicisimpins B (**147**)	*Penicillium simplicissimum MA-332*	*A. hydrophilia* (MIC 32 µg/mL)
		*E. coli* (MIC 32 µg/mL)
		*M. luteus* (MIC 64 µg/mL)
		*P. aeruginosa* (MIC 32 µg/mL)
		*V. alginolyticus* (MIC 32 µg/mL)
		*V. harveyi* (MIC 16 µg/mL)
		*V. parahaemolyticus* (MIC 32 µg/mL)
		*C. gloeosprioides* (MIC 16 µg/mL)
Penicisimpins C (**148**)	*Penicillium simplicissimum MA-332*	*A. hydrophilia* (MIC 16 µg/mL)
		*E. coli* (MIC 8 µg/mL)
		*M. luteus* (MIC 16 µg/mL)
		*P. aeruginosa* (MIC 8 µg/mL)
		*V. alginolyticus* (MIC 16 µg/mL)
		*V. harveyi* (MIC 8 µg/mL)
		*V. parahaemolyticus* (MIC 8 µg/mL)
		*C. gloeosprioides* (MIC 8 µg/mL
Citreoisocoumarin (**149**)	*Penicillium vinaceum*	*S. aureus* (Zone of inhibition 19 mm at 100 µg/disk)
Nucleosides		
Rocheicoside A (**150**)	*Streptomyces rochei 06CM016*	*E. coli* O157:H7 (MIC 16 µg/mL)
		MRSA DSM (MIC 8 µg/mL)
		*C. albicans* DSM (MIC 4 µg/mL)
Plicacetin (**151**)	*Streptomyces rochei 06CM016*	*E. coli* O157:H7 (MIC 8 µg/mL)
		MRSA DSM (MIC 4 µg/mL)
		*C. albicans* DSM (MIC 8 µg/mL)
Norplicacetin (**152**)	*Streptomyces rochei 06CM016*	*E. coli* O157:H7 (MIC 16 µg/mL)
		MRSA DSM (MIC 8 µg/mL)
		*C. albicans* DSM (MIC 4 µg/mL)
*p*-amino benzamido uracil (**153**)	*Streptomyces rochei 06CM016*	*E. coli* O157:H7 (MIC 16 µg/mL)
		MRSA DSM (MIC 16 µg/mL)
		*C. albicans* DSM (MIC 8 µg/mL)
Cytosamine (**154**)	*Streptomyces rochei 06CM016*	*E. coli* O157:H7 (MIC 16 µg/mL)
		MRSA DSM (MIC 16 µg/mL)
		*C. albicans* DSM (MIC 8 µg/mL)
Miscellaneous Compounds		
Terretrione A (**151**)	*Penicillium vinaceum*	*C. albicans* (Zone of inhibition 27 mm at 100 µg/disk)
Terretrione C (**152**)	*Penicillium* sp. *CYE-87*	*C. albicans* (MIC 32 µg/mL)
Terretrione D (**153**)	*Penicillium* sp. *CYE-87*	*C. albicans* (MIC 32 µg/mL)
α-Cyclopiazonic acid (**154**)	*Penicillium vinaceum*	*E. coli* (Zone of inhibition 20 mm at 100 µg/disk)
5-methoxydihydrosterigmato-cystin (**155**)	*Aspergillus versicolor*	*S. aureus* (MIC 13 µg/mL)
		*B. subtilis* (MIC 3 µg/mL)
Caerulomycin A (**156**)	*Actinoalloateichus cyanogriseus*	*C. albicans* (MIC 0.8–1.6 µg/mL)
		*C. albicans* CO9 (MIC 0.8–1.6 µg/mL)
		*C. glabrata* HO5^FlucR^ (MIC 0.4–0.8 µg/mL)
		*C. krusei* GO3^FlucR^ (MIC 0.8–1.6 µg/mL)
Trichodin A (**157**)	*Trichoderma* sp.	*B. subtilis* (IC_50_ 27 µM)
		*S. epidermidis* (IC_50_ 24 µM)
		*C. albicans* (IC_50_ 25 µM)
Pyridoxatin (**158**)	*Trichoderma* sp.	*B. subtilis* (IC_50_ 4 µM)
		*S. epidermidis* (IC_50_ 4 µM)
		*S aureus* (MRSA), (IC_50_ 4 µM)
		*C. albicans* (IC_50_ 26 µM)
		*T. rubrum* (IC_50_ 4 µM)
1-(10-Aminodecyl) Pyridinium (**159**)	*Amycolatopsis alba*	*B. subtilis* NCIM 2439 (MIC > 70 µg/mL)
		*B. pumilus* NCIM 2327 (MIC > 90 µg/mL)
		*S. aureus* NCIM 5021 (MIC > 160 µg/mL)
		*A. formicans* NCIM 2319 (MIC > 150 µg/mL)
		*E. coli* NCIM 2067 (MIC > 90 µg/mL)
Porric acid D (**160**)	*Alternaria* sp.	*S. aureus* (MIC 100 µg/mL)
Altenusin (**161**)	*Alternaria* sp.	*S. aureus* (MIC 25 µg/mL)
*p*-Hydroxybenzoic acid (**162**)	*Pseudoalteromonas flavipulchra*	*V. anguillarum* (MIC 1.25 mg/mL)
*trans*-Cinnamic acid (**163**)	*Pseudoalteromonas flavipulchra*	*V. anguillarum* (MIC 1.25 mg/mL)
*N*-hydroxybenzoisoxazolone (**164**)	*Pseudoalteromonas flavipulchra*	*V. anguillarum* (MIC 0.25 mg/mL)
Bacilosarcin B (**165**)	*Bacillus subtilis*	*S. aureus* (MIC 4 µM)
amicoumacin A (**167**)	*Bacillus subtilis*	*S. aureus* (MIC 19 µM)
		*B. subtillis* (MIC 19 µM)
Microluside A (**169**)	*Micrococcus* sp. *EG45*	*E. faecalis* JH212 (MIC 10 µM)
		*S. aureus* NCTC 8325 (MIC 13 µM)
